# High‐Entropy Oxides for Rechargeable Batteries

**DOI:** 10.1002/advs.202401034

**Published:** 2024-04-22

**Authors:** Biao Ran, Huanxin Li, Ruiqi Cheng, Zhaohui Yang, Yi Zhong, Yonghong Qin, Chao Yang, Chaopeng Fu

**Affiliations:** ^1^ School of Materials Science and Engineering, Shanghai Key Laboratory of Advanced High‐temperature Materials and Precision Forming Shanghai Jiao Tong University Shanghai 200240 China; ^2^ Physical & Theoretical Chemistry Laboratory, Department of Chemistry University of Oxford South Parks Road Oxford OX1 3QZ UK; ^3^ Faculty of Materials Science and Energy Engineering/Institute of Technology for Carbon Neutrality, Shenzhen Institute of Advanced Technology Chinese Academy of Sciences Shenzhen 518055 China

**Keywords:** configuration entropy;high‐entropy oxides;ionic conductivity;structural stability

## Abstract

High‐entropy oxides (HEOs) have garnered significant attention within the realm of rechargeable batteries owing to their distinctive advantages, which encompass diverse structural attributes, customizable compositions, entropy‐driven stabilization effects, and remarkable superionic conductivity. Despite the brilliance of HEOs in energy conversion and storage applications, there is still lacking a comprehensive review for both entry‐level and experienced researchers, which succinctly encapsulates the present status and the challenges inherent to HEOs, spanning structural features, intrinsic properties, prevalent synthetic methodologies, and diversified applications in rechargeable batteries. Within this review, the endeavor is to distill the structural characteristics, ionic conductivity, and entropy stabilization effects, explore the practical applications of HEOs in the realm of rechargeable batteries (lithium‐ion, sodium‐ion, and lithium‐sulfur batteries), including anode and cathode materials, electrolytes, and electrocatalysts. The review seeks to furnish an overview of the evolving landscape of HEOs‐based cell component materials, shedding light on the progress made and the hurdles encountered, as well as serving as the guidance for HEOs compositions design and optimization strategy to enhance the reversible structural stability, electrical properties, and electrochemical performance of rechargeable batteries in the realm of energy storage and conversion.

## Graphical abstract

The review furnishes the evolving landscape of high‐entropy oxides (HEOs)‐based cell component materials, including anode, cathode, electrolytes, and electrocatalysts. This sheds light on the progress made and the hurdles encountered, guiding the HEOs compositions design and optimization to enhance the reversible structural stability and electrochemical performance of rechargeable batteries in the realm of energy storage and conversion.

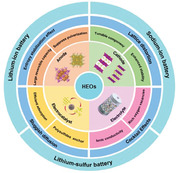

## 1 Introduction

The escalating demand for energy underscores the urgency to develop sustainable and clean energy sources as viable alternatives to fossil fuel technologies.^[^ ^1^ ^]^ Rechargeable batteries interconvert electrical power and chemical internal energy, and effectively overcome the fluctuation of renewable energy.^[^ ^2^ ^]^ Consequently, the development of large‐scale and low‐cost energy storage stations has become urgent, and the initiative research on high‐performance electrode materials and thorough clarification of structural features have become more and more important.^[^ ^3^ ^]^ Despite great efforts have been made to explore economical and efficient materials, a steady stream of considerable problems have emerged in the storage and operation of rechargeable batteries, such as the structural stability and adaptability to the environment.^[^ ^3^ ^]^ It is imperative to implement enormous endeavors to develop superior electrode materials and energy storage systems.^[^ ^4^, ^5^ ^]^

**Figure 1:**
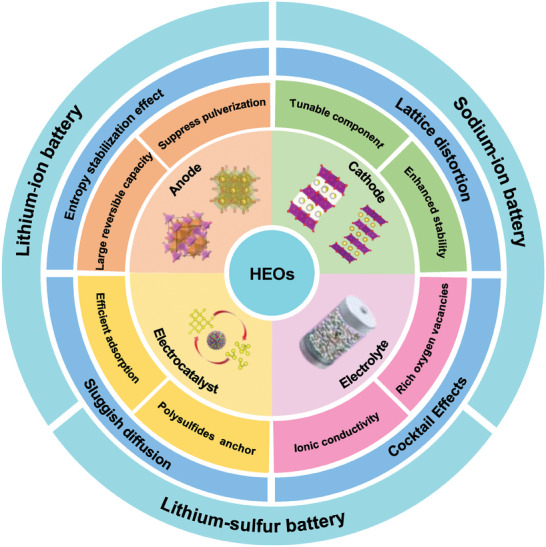
Schematic diagram of HEOs applications in rechargeable batteries.

Initially, high entropy alloys (HEAs) were proposed in 2004 to investigate uncharted regions of the metal alloy phase diagram. These alloys typically consist of more than five primary metal elements, with the atomic percentage ratio typically falling within the range from 5% to 35%.^[^ ^6^ ^]^ Subsequently, the high entropy concept spreads rapidly to the ministry of ceramic materials,^[^ ^7^ ^]^ such as oxides,^[^ ^8^ ^]^ nitrides,^[^ ^9^ ^]^ sulfides,^[^ ^10^ ^]^ phosphides,^[^ ^11^ ^]^ silicides,^[^ ^12^ ^]^ etc. High‐entropy oxides (HEOs) are the representative emerging class of entropy‐stabilized single‐phase compounds consisting of a diverse range of cation species and oxide anions, which exhibit a wide variety of structural forms and offer great compositional flexibility, high structural stability, superionic conductivity, capably modulate the electrochemical properties.^[^ ^13^ ^]^ Hence, the unique characteristics of HEO materials are widely recognized as promising contenders for vital components in rechargeable batteries, spanning roles in the anode, cathode, electrolyte, and electrocatalysts for lithium‐ion batteries (LIBs), sodium‐ion batteries (SIBs), and lithium‐sulfur (Li–S) batteries (**Figure** **1**).^[^ ^14^ ^]^

The distinctive structure and diverse functional properties of HEOs, encompassing superior physicochemical stability and tunable electrochemical performance, have generated significant academic interest in the realm of rechargeable battery applications. Recent works reviewed the fundamental aspects and electrochemical properties of HEOs,^[^ ^13c^ ^]^ and summarized the applications in energy storage and conversion.^[^ ^13b^ ^]^ For example, Anselmi‐Tamburini et al. discussed the effect of configuration entropy on the phase stability of HEOs.^[^ ^16a^ ^]^ Hahn et al. shared the uniqueness of HEOs by the view of entropy, enthalpy, and synergy.^[^ ^15^ ^]^ Wu's group summarized the recent developments and challenging perspectives of HEOs for chemical catalysis.^[^ ^16b^ ^]^ Lei et al. summarized HEOs as the anode materials for LIBs only,^[^ ^17^ ^]^ and Yan et al. reviewed recent advances in high‐entropy layered cathodes for SIBs,^[^ ^18a^ ^]^ Zanin et al. presented and compared these strategies to designer medium‐ and high‐entropy materials for Li–S batteries.^[^ ^19b^ ^]^ Dou et al. analyze the effects of elements, structure, and morphology on the properties of high‐entropy materials in lithium‐based rechargeable batteries.^[^ ^20^ ^]^ Despite the brilliance of HEOs in energy conversion and storage applications, there is still lacking a comprehensive review for both entry‐level and experienced researchers that succinctly encapsulates the present status and the challenges inherent to HEOs.

In this review, we provide a comprehensive overview on HEOs in terms of fundamental concepts, structure characteristics, ionic conductivity, entropy stabilization effects, synthesis methods, and various distinctive component applications in the field of rechargeable batteries. We comprehensively compare the advantages of various synthesis methods, and analyze the influence of elements, structure, and morphology of HEOs on ionic conductivity, reaction kinetics, and cyclic stability. Additionally, we explore the potential of density functional theory (DFT) calculations and Machine Learning to expedite the design process for HEOs. The review aims to provide guidance and strategy for the design of HEO components, enhancement of corresponding electrochemical properties, and infusing new momentum into the development of high‐performance batteries.

## 2 Concept, Structures, and Properties of HEOs

### 2.1 Fundamental Concept

HEO is a single‐phase oxide created through the solid solution of five or more metal elements within the same sub‐lattice. The increase of constituent elements improves the configuration entropy of the whole solid solution system. When the configuration entropy is greater than 1.5 R, stable single‐phase crystals are likely to appear under high‐temperature sintering conditions, which can be also called entropy‐stable oxides. Distinguishing from the traditional doped oxide, HEOs have the representative four core effects, including large configurational entropy, sluggish diffusion, lattice distortion, and cocktail effects.^[^ ^12^, ^21^ ^]^ To understand the definition of HEOs and calculate their configuration entropy, a canonical definition was proposed for high entropy compounds, and the configurational entropy of the whole system can be calculated by the following Equation (1). Since the cations play a dominant role in the conformational entropy of HEOs, Equation (1) can also be transformed into Equation (2) owing to oxygen anions along in HEOs. This transformation stems from the negligible contribution of oxygen anions to the configurational entropy in HEOs. In a 5‐cation system, the configurational entropy peaks when all cations are equimolar, with the maximum *S*_config_ value reaching 1.61 R:^[^ ^13^, ^15^ ^]^

(1) $$S_{conf}=-R\left\{{\left(\sum_{a=1}^{M}x_{a}lnx_{a}\right)}_{cation-site}+{\left({\sum_{b=1}^{N}y_{b}lny}_{b}\right)}_{anion-site}\right\}$$

(2) $$S_{HEO-conf}=-R\left(\sum_{a=1}^{M}x_{a}lnx_{a}\right)cation-site$$

where *R* represents the ideal gas constant and *x_a_*, *y_b_* being the mole fraction of the corresponding elements in the cation‐site and anion‐site, respectively.^[^ ^22^ ^]^

It can be classified according to the value of entropy configuration, the component system with *S*_config_  ≥  1.5 R can be considered as “high entropy oxides,” the component system with 1 R <  *S*_config_ <  1.5 R can be identified as “medium entropy oxides,” and the component system with *S*_config_ < 1 R as the “low entropy oxides.” High configuration entropy can increase the compatibility between main components, reduce the total free energy, and avoid phase separation forming intermetallic compounds. It is the decisive factor for the HEOs system to achieve phase stability at high temperatures.^[^ ^16a^ ^]^

### 2.2 Classification and Structure

**Figure 2:**
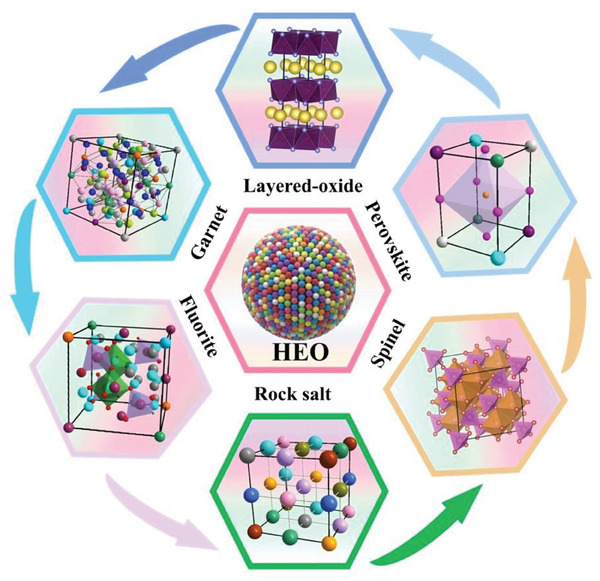
Typical structures of HEOs.

The emerging of HEOs provides more opportunities to tune properties and burst the shackles of design regime, on account that the crystal structures of HEOs are more diverse compared with the simple face‐centered cubic, body‐centered cubic, and hexagonal close‐packed structures in HEA.^[^ ^23^ ^]^ Achieving a comprehensive grasp of their diverse depends on a methodical classification and comparative assessment of their structural features, rooted in the spatial arrangements of poly‐metallic atoms. HEOs represent these compounds that have the oxygen atom to build the cation lattice framework as different crystalline structures and then have various metallic cations to sit in the octahedral or tetrahedron sites in a single‐phase crystal structure (**Figure** **2**), including rock salt,^[^ ^24^ ^]^ fluorite,^[^ ^25^ ^]^ perovskite,^[^ ^26^ ^]^ spinel,^[^ ^27^ ^]^ garnet,^[^ ^28^ ^]^ and layered HEO structures.^[^ ^29^ ^]^ The diversity of HEOs crystal structures provides a strategy for discovering new crystal substances, untapped opportunities for performance optimization, and more possibilities for the regulation of energy band and chemical bond changes.^[^ ^30^ ^]^

Rock salt is a single‐phase crystalline structure, of which the anions are located at the corner tops and the cubical face centers, while the cations fill the octahedral voids densely packed. The rock‐salt (F𝑚3⎯𝑚) type oxides are the predominant member of the HEO family, contain only one Wyckoff‐site cation, and belong to the isometric six‐octahedral crystal class and AX‐type compounds.^[^ ^13c^ ^]^ The first reported rock‐salt HEO (Mg_0.2_Co_0.2_Ni_0.2_Cu_0.2_Zn_0.2_)O, possessing a relatively simple and stable structure with divalent state metal cations, and the configuration entropy was 1.61 R, achieved reversible transformation from multiphase to single‐phase.^[^ ^7^ ^]^

Compared with rock‐salt structure, spinel‐HEOs enrich the chemical valence state types and capably accommodate trivalent ions, which are regarded as the more competitive candidates for electrode materials.^[^ ^31^ ^]^ The spinel structure is more promising by the two kinds of different Wyckoff sites for cation and unique 3D ion transport pathways. Typically, spinel oxide (AB_2_O_4_) adopts a crystal structure where divalent metal cations occupy the tetragonal (A) site, while trivalent metal cations in the octahedral (B) site, thereby forming a close‐packed cubic arrangement. The tetragonal and octahedral sites can accommodate various types of metal cations synchronously. The distinctive crystal structure allows for the presence of trivalent ions, consequently leading to an increase in the range of valence changes and two folders of configuration entropy compared with rock‐salt structure, in which the additional site can increase the range of valence states electrochemical Faraday reaction process.

High‐entropy fluorite oxides (HEFOs) are those solid solution compounds where ZrO_2_, CeO_2,_ and HfO_2_ are the basic framework, and including additional metal elements, such as La, Y, Ti, Gd, Mg, Yb, and Ca to stabilize the fluorite structure. These types of HEFOs materials exhibit a cubic Fm‐3m space group and possess prime properties such as high hardness and melting temperatures, high ionic or mixed conductivities,^[^ ^25^ ^]^ and low thermal conductivities.

High entropy layered oxide structures, such as O3,^[^ ^32^ ^]^ and P2‐type,^[^ ^33^ ^]^ have been utilized as the cathodes for SIBs, of which five or more metal cations are jointly sharing the octahedral transition metal (TM) site in the transition metal oxide (TMO) slabs, and Na occupying in the octahedral site or prismatic site. These metal cations can provide charge compensation for capacity, serve as the entire structural host, and raise the average voltage.^[^ ^34^ ^]^ The high entropy stabilization effect enables layer‐structure cathode materials with superior structure stability and improved storage capacity.^[^ ^35^ ^]^

High‐entropy perovskite oxides (HEPOs) exhibit unique physical properties and highly tunable chemistries, band gap structure by substituting different metal cations, which have been widely used in solar and fuel cells,^[^ ^36^ ^]^ electrode materials,^[^ ^26^ ^]^ proton conductors,^[^ ^37^ ^]^ dielectric materials.^[^ ^38^ ^]^ Typically, the perovskite structure can be adjusted by the ratio of the cation and oxygen radii, and exhibit unique multiferroic properties and electronic configurations. These phenomena arise from the interplay between the local bonding geometry and the cation interactions at different coordination environment sites. Moreover, the presence of corner‐sharing oxygen octahedral units allows for a high degree of freedom in manipulating the structure of perovskite materials.^[^ ^39^ ^]^

There are other kinds of structures apart from the crystal structures mentioned above, including pyrochlore,^[^ ^40^ ^]^ bixbyite,^[^ ^41^ ^]^ rutile,^[^ ^42^ ^]^ and CaF_2_‐type.^[^ ^43^ ^]^ Due to the unique high entropy effects, the above‐mentioned HEOs have superior phase stability, distinctive electrical conductivity, and magnetic versatility compared with the analogous structures of binary or ternary metal oxides.

### 2.3 Entropy Stabilization Effect

The entropy stabilization effect is one of the most distinguishing features of HEOs.^[^ ^44^ ^]^ Entropy is the main driving force that is responsible for the formation of single‐phase solid solutions. HEOs exhibit the presence of intermediate anions that separate adjacent cations, which can suppress short‐range ordering and increase the configuration entropy. HEOs are typically entropy‐stable materials with superior phase stability, structural and functional versatility, and tunability.^[^ ^16^ ^]^ Its crystal structure with stable entropy shows a reversible transition between single‐phase and multiphase structures at elevated temperatures.^[^ ^15^ ^]^ Since the configurational entropy of oxygen anions in the HEO solid solution is ≈0, the whole entropy is approximately the sum of cation entropy. According to the Gibbs free energy equation Δ *G*_mix_ =  Δ*H*_mix_ − *T*Δ*S*_mix_, to improve the system stabilization and maximize the Δ*S*_mix_value, the large configuration entropy in HEOs is considered to increase the solid solubility of polymetallic components, which can be perceived to optimize the candidate properties.^[^ ^13b^ ^]^ In thermodynamics, reactions with significantly negative free energy are considered the most favorable. The driving force for the formation of single‐phase HEOs comes from the uniformity of multiple elements generating high configurational entropy. To harness this entropic driving force and promote the HEOs formation, elevated temperatures are typically necessary to maximize the impact of the *TΔS* term in the Gibbs free energy equation. Upon reaching the desired state, rapid quenching is often employed to stabilize the phase.^[^ ^45^ ^]^ A series of subsequent reports have confirmed the entropy‐controlled transition from the multiphase to the single‐phase state, the phase transition is heat‐absorbing and reversible at an elevated temperature. The Gibbs free energy of transformation is the most crucial term in determining solubility when mixing non‐isostructural components. The stabilization effect of large configuration entropy for HEOs has been demonstrated with a significant benefit on the retention of storage capacity and improved structural stability.^[^ ^46^ ^]^

### 2.4 Ionic Conductivity

The ionic conductivity of HEOs is a critical property that determines their potential applications in solid‐state electrolytes, fuel cells, sensors, and other electrochemical devices.^[^ ^13b^ ^]^ HEOs represent a class of materials characterized by multiple cations occupying the same lattice site. They demonstrate outstanding electron conductivity and ion diffusion rates, facilitated by abundant oxygen vacancies. These oxygen vacancies create ample transport pathways for charge carriers, thereby enhancing both ionic and electronic conductivity, and ultimately improving overall conductivity.^[^ ^47^ ^]^ Moreover, the presence of multi‐variable valence metal cations contributes to achieving both reversibility and accessibility of charge compensation. Additionally, the introduction of oxygen vacancies can significantly enhance ion diffusion and charge transfer processes.^[^ ^48^ ^]^ It is crucial to highlight that the electrical properties in HEOs can be finely tuned by adjusting the electronic structure and band gaps, a feat accomplished by judiciously selecting suitable constituent elements. HEOs with more than five principal components offer an extensive chemical space for regulating electronic configuration and improving conductivity.^[^ ^49^ ^]^ For instance, the electrical transport mechanism in HEOs has been reported to partially switch from electronic to ionic, leading to a remarkable increase in conductivity upon the incorporation of Li.^[^ ^50^ ^]^

The following strategies are generally adopted to modify HEOs to improve the ionic conductivity, including 1) doping electronegative elements into the HEO lattice to reduce the ion migration barrier and improve the ionic conductivity. For example, Ma et al. introduced Li into the spinel HEO to obtain the (FeMgNiCrMnLi)_3_O_4_ anode, inducing the growth of oxygen vacancies and regulating the Li^+^ ion intercalation process.^[^ ^51^ ^]^ 2) Designing nanostructure: The synthesis of nanostructured HEO materials is undertaken to leverage the large specific surface area and shortened ion transport path inherent in nanostructured materials, which effectively enhances the ion conduction rate.^[^ ^52^ ^]^ 3) Construction of heterostructure: Incorporating an additional distinct structure into the composites to form a heterostructure for optimizing the ionic conductivity performance.^[^ ^53^ ^]^ 4) Creating rich oxygen vacancies.^[^ ^54^ ^]^

The oxygen vacancies are of great significance for the ion conductivity in HEOs as follows. i) The unbalanced charge distribution around oxygen vacancies will create foreign coulomb force and accelerate the migration of electrons and ions.^[^ ^55^ ^]^ ii). The oxygen defects derived from the lattice distortion facilitate surface ion adsorption and improve reaction kinetics as well as decrease the charge migration carrier. Nicolas et al. clarified the content of lithium‐substituted HEOs affecting the concentration of oxygen vacancies in (MgCoNiCuZn)_1−_ *_x_* Li*_x_* O_1−_ *_y_* and the valence state of cations through charge compensation mechanisms. The sample reaches quite large Li^+^ ionic conductivity values with *y* ≈ 0.05 at *x* = 0.3, and a lower activation energy.^[^ ^56^ ^]^ Through finely tuning the oxygen stoichiometry, and then adjusting the distribution and concentration of oxygen vacancies to achieve higher ionic conductivity for HEOs.

## 3 Synthetic Methods

### 3.1 Solid‐State Sintering

**Figure 3:**
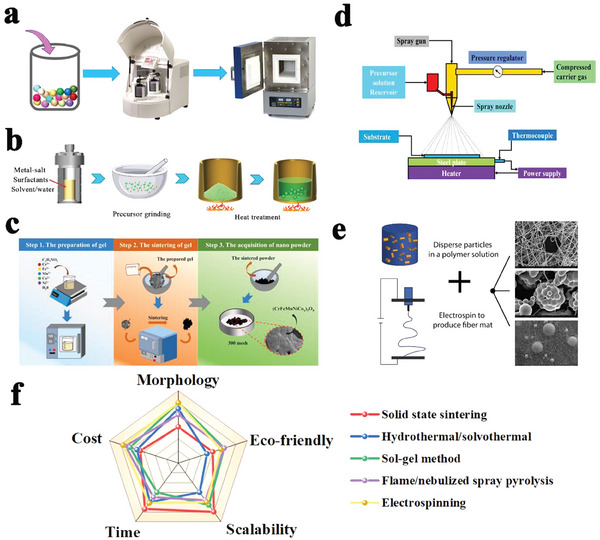
a) Solid‐state sintering method. Reproduced with permission.^[^ ^48b^ ^]^ Copyright 2021, Springer. b) Hydrothermal/solvothermal method. Reproduced with permission.^[^ ^59b^ ^]^ Copyright 2022, Wiley‐VCH. c) Sol‐gel method. Reproduced with permission.^[^ ^60^ ^]^ Copyright 2023, Elsevier. d) Flame spray and nebulized spray pyrolysis. Reproduced with permission.^[^ ^61^ ^]^ Copyright 2022, Elsevier. e) Electrospinning. Reproduced with permission.^[^ ^62^ ^]^ Copyright 2018, Elsevier. f) Comparison of various synthetic methods.

The solid‐state sintering method is one of the most conventional synthesis methods for HEOs, which is an uncomplicated experimental procedure and facile operation for industrialized production.^[^ ^57^ ^]^ The ordinal synthesis steps are shown in **Figure** **3**a, five or more kinds of metallic oxides are mixed in stoichiometric proportion with ball milling, compaction, and annealing at high temperature in air to obtain the single‐phase structure HEOs. Among the preparation factors, processing temperature, mixing time, heating and cooling rates are the critical factors to generating the desired single‐phase HEO particles, otherwise leading to the undesired phase separation caused by non‐uniform distribution of metal elements, and the nanocrystalline overgrowth.^[^ ^58^ ^]^ Nevertheless, the synthesis step is relatively simple, and controllable composition for HEOs, there are some shortcomings, such as it requires a long‐term sintering time at high temperatures, undesirable presence of agglomerations, and difficult to obtain nano‐structured particles with regular morphology. For example, Wang et al. synthesized the spinel‐structured HEO (FeCoNiCrMn)_3_O_4_ particles by mixing equimolar amounts of metal oxides though a planetary ball milling, and then subjected to calcination under 900 °C for 12 h.^[^ ^48^ ^]^

### 3.2 Solvothermal Synthesis

The solvothermal techniques are simple, efficient, and morphologically controllable methods for preparing nanoparticles.^[^ ^63^ ^]^ As shown in Figure 3b, Solvothermal methods take stoichiometric proportion metal salts dissolved in deionized water or other solvents as the reaction precursor, fully stirring to form a uniform solution, and trigger chemical reactions in an autoclave under high pressure and temperature conditions, the final HEO products can be obtained after vacuum drying overnight. Compared with the solid‐state method, the samples prepared by the solvothermal method have a uniform spherical shape, and lower sintering temperature to synthesize single‐phase HEOs with uniform particle dispersion.^[^ ^59^ ^]^ For example, Wang et al. fabricated a single‐phase spinel structure HEO (CoCuFeMnNi)_3_O_4_ by solvothermal method at 400 °C for 2 h, the average size of the final particles is about 5 nm.^[^ ^64^ ^]^ Additionally, the solvothermal techniques allow the formation of well‐defined crystalline structure, controlled particle size, and morphology structure by precisely regulating processing parameters such as temperature, pressure, reaction time, the concentration of precursors and additives, and pH value.^[^ ^65^ ^]^ However, it requires a relatively complicated experimental process, the use of toxic solvents, and post‐treatment steps to remove impurities. To achieve large‐scale production suitable for electrodes, the experimental conditions need to be further optimized while ensuring homogeneous mixing of these elements.

### 3.3 Sol‐Gel Synthesis

The sol‐gel method is often used to synthesize HEOs, and contains three primary processing steps (Figure 3c). First, it involves the formation of multi‐components sol, which is a colloidal suspension of nanoparticles in the liquid medium, followed by gelation and subsequent heat treatment in air, the refined HEO particles obtained by mashing and grinding. The multi‐component precursor solutions can be easily modified by adjusting the concentrations and ratios of the starting materials, enabling the synthesis of a wide range of compositions, especially containing non‐equimolar heterogeneous atoms components.^[^ ^60^ ^]^ Additionally, the sol‐gel method makes it possible for the incorporation of dopants or additives to tailor the morphology and structure of HEO products. In contrast to conventional solid‐state sintering, the sol‐gel method offers significant advantages, including lower calcination temperatures and the production of particles with a more uniform size distribution and finer morphology.^[^ ^66^ ^]^ Researchers have used the sol‐gel method to prepare various structure types of HEOs. For example, Beatrix et al. reported the synthesis of a high entropy spinel oxide, (Cr_0.2_Mn_0.2_Fe_0.2_Co_0.2_Zn_0.2_)_3_O_4_, with the sol‐gel method.^[^ ^67^ ^]^ They mixed the precursor solutions of the stoichiometric constituent metal ions, followed by heating and annealing to obtain the desired spinel structure. Tang et al. employed the sol‐gel method to prepare a high‐entropy perovskite oxide, (La_0.6_Sr_0.4_)(CoFeMnNiMg)O_3_.^[^ ^68^ ^]^ In general, the sol‐gel method has shown great potential for the synthesis of HEOs, providing a controlled composition, morphology, and properties. Further research in this area is expected to explore novel compositions and optimize the synthesis parameters to enhance the performance of HEO materials.

### 3.4 Flame Spray and Nebulized Spray Pyrolysis

Flame spray pyrolysis (FSP) and nebulized spray pyrolysis (NSP) are two commonly used industrial technologies for atomizing the precursor solution to prepare HEOs.^[^ ^61^ ^]^ As depicted in Figure 3d, the precursor solution is atomized into fine droplets using compressed carrier gas, and collected, heat treated on the steel plate. This technique can regulate the size of synthesized HEO particles by adjusting various processing parameters such as temperature, precursor concentration, and carrier/nebulizer flow rate. For example, Sarkar et al. synthesized (CoCuMgNiZn)O homogenous nanocrystalline particles with these two spray pyrolysis techniques, reduced the synthesis time, and stabilized the metastable phase.^[^ ^69^ ^]^ There are some similarities involving the use of pyrolysis to synthesize oxide nanoparticles between the two techniques. Accompanied by the fast spray process, both FSP and NSP shorten the processing period, and obtain a stable compound particle with uniform size, which exhibits a bright industrial application prospect on account of the high‐efficiency yield characteristics.^[^ ^70^ ^]^

### 3.5 Electrospinning

Electrospinning technology is a versatile and efficient method for producing HEO nanofibers. Electrospinning allows the production of HEO nanofibers with controlled morphology and composition, including their diameter, alignment, and surface area.^[^ ^71^ ^]^ Such as Liu et al. utilized the electrospinning technology to fabricate the HEO CoZnCuNiFeZrCeO*_x_* sub‐1 nm nanowires with highly ordered structures under facile conditions.^[^ ^72^ ^]^ This control allows for tailored properties and improved electrochemical performance, which makes it a promising technique for synthesizing HEOs with unique properties. However, limitations in complex composition control and process parameters complexity should be considered for large‐scale preparation.^[^ ^73^ ^]^

Above all, the choice of suitable preparation methods for HEOs depends on their specific demands and purpose. The technical characteristics of these synthetic methods are summarized in Figure 3f. High‐temperature solid‐state and ball milling mixing is the preferred method for commercial large‐scale preparation. Because it is convenient to directly control the chemical composition of raw materials, processing steps are simple, without post‐treatment of harmful solvents. Sol‐gel method is advantageous for more complex ingredient preparation and morphology control, especially in cases where ultra‐temperature stability is required. The hydrothermal/solvothermal method shows unique advantages and is more suitable for constructing special morphology and controlling the uniformity of components. Furthermore, the shape of HEOs also affects experimental results, leading to more complex preparation processes using the aforementioned methods in actual productions.

## 4 Applications of Rechargeable Batteries

Ever since the discovery of Li or Na substituted (MgCoNiCuZn)O with superionic conductivity, there has been a continuous surge of innovative electrochemical storage applications centered around HEO materials.^[^ ^74^ ^]^ Owing to the vast element space, multi‐electron redox mechanism, adjustable electrochemical properties, and unique crystal structure retention ability, HEOs are gradually becoming the focus for various rechargeable batteries, including LIBs,^[^ ^17^ ^]^ SIBs,^[^ ^75^ ^]^ and Li–S batteries.^[^ ^19^ ^]^ In this section, we highlight the advantages and comprehensively summarize the research progress and modification strategies of HEOs component materials for rechargeable batteries, which open a new avenue for advanced electrode materials development.

### 4.1 Anode

**Figure 4:**
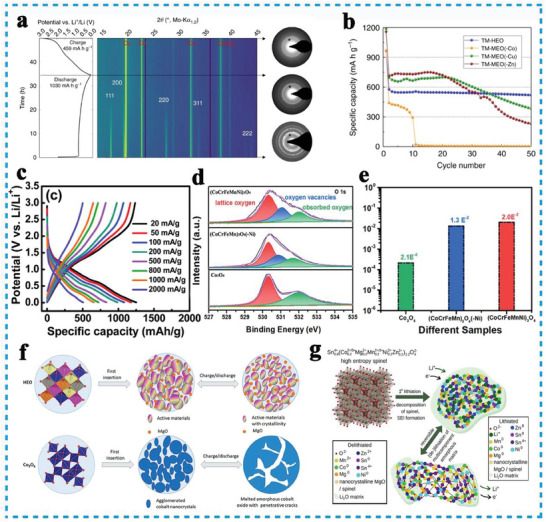
a) The operando XRD results on HEO during the first full lithiation/delithiation cycle; b) the comparison of cycling performance between HEO and the 4‐cation systems, Reproduced with permission.^[^ ^8^ ^]^ Copyright 2018, Springer Nature. c) Charge‐discharge curves of the spinel electrode were measured at various rates. Reproduced with permission.^[^ ^52^ ^]^ Copyright 2020, RSC. d) Evaluate the oxygen vacancies in HEO; and e) electrical conductivity. Reproduced with permission.^[^ ^66b^ ^]^ Copyright 2021, American Chemical Society. f) Comparation of Lithiation/delithiation mechanisms for HEO and cobalt oxide electrodes. Reproduced with permission.^[^ ^77b^ ^]^ Copyright 2019, Elsevier. g) Schematic of Li‐storage mechanism for the Sn_0.8_(Co_0.2_Mg_0.2_Mn_0.2_Ni_0.2_Zn_0.2_)_2.2_O_4_ conversion‐alloying anode material. Reproduced with permission.^[^ ^78^ ^]^ Copyright 2022, American Chemical Society.

Since Rost et al. reported the entropy‐stabilized rocksalt HEOs in 2015, providing new possibilities to overcome these limitations in the conversion type electrode materials. Sarkar initially reported the reversible Li‐ion storage properties of the rock‐salt (Co_0.2_Cu_0.2_Mg_0.2_Ni_0.2_Zn_0.2_)O anode, benefited from the entropy stabilization effect, the remained (200) lattice plane in HEO indicating the conservation of the original rock‐salt structure during the entire (de)lithiation process (**Figure** **4**a). It also exhibited excellent cycle stability compared to the 4‐cation oxides (Figure 4b).^[^ ^8^ ^]^ In contrast, spinel‐structured HEOs have two different cation Wyckoff sites and 3D Li^+^ diffusion paths, distinct structures with hybrid valence, variant element radii, and show outstanding electrochemical performance.^[^ ^48^ ^]^ Chen et al. first reported a new kind of spinel (Mg_0.2_Ti_0.2_Zn_0.2_Cu_0.2_Fe_0.2_)_3_O_4_ anode material containing heterovalent cations (Fe^3+^, Ti^4+^), which enriched the spinel‐HEOs diversities and delivered a large reversible capacity of 504 mAh g^−1^ (rate:100 mA g^−1^) after 300 cycles.^[^ ^57b^ ^]^ Nguyen et al. also investigated non‐equimolar high entropy spinel anode, of which Cr, Mn, Fe, Co, and Ni are evenly distributed with different valence states, contributing larger configuration entropy.^[^ ^76^ ^]^ Without dummy MgO structural pillars, the spinel HEOs anode still provided exceptional redox stability and outstanding capacity retention at various current rates (Figure 4c).^[^ ^66b^ ^]^ To deal with poor electrical conductivity and relieve volume expansion in practical application, Zhao et al. demonstrated that Ni determining spinel‐HEO (Co_0.2_Cr_0.2_Fe_0.2_Mn_0.2_Ni_0.2_)_3_O_4_ with fruitful oxygen vacancies (Figure 4d), these oxygen vacancies provided extra Li^+^ ion accommodation sites to accelerate the Li^+^ migration (Figure 4e).^[^ ^59b^ ^]^ Qiu et al. revealed the essence that uniformly dispersed active metal cations combined with inert MgO pillars would significantly improve the cycle stability (figure 4f).^[^ ^77b^ ^]^ To ensure stable electrochemical performance for multicomponent solid solution‐type material, Moździerz et al. proposed Sn‐based Sn_0.8_(Co_0.2_Mg_0.2_Mn_0.2_Ni_0.2_Zn_0.2_)_2.2_O_4_ spinel HEO conversion‐alloying anode material (Figure 4g). Owing to the reversible reactivity of the conversion‐alloying HEOs matrix, and kept the cations in high disorder at the atomic scale, exhibited a large specific capacity above 600 mAh g^−1^ at a current density of 50 mA g^−1^ and good long cycle retention for 500 cycles.^[^ ^78^ ^]^ The remarkable Li storage performance of HEOs is attributed to their unique composition, comprising five or more different functional components in a single‐phase solid solution without short‐range order. This configuration leads to a high configurational entropy, which in turn stabilizes the crystal structure.

Understanding the energy storage mechanism of HEOs involves a comprehensive evaluation of their structural, electronic, and electrochemical properties. The theoretical capacities of conversion‐type HEOs anode materials are directly related to the number of electrons transferred by its molecular formula unit. The combination of different metal cations in HEOs generates synergistic effects that influence the energy storage mechanism in LIBs. The interaction between the metal cations and lithium ions, as well as the modification of electronic structure during lithiation/delithiation process, resulted in unique energy storage behaviors that differ from single‐component oxides.^[^ ^77b^ ^]^ Therefore, it is essential to figure out the selection rule of different elements and identify the potential roles of various elementals for HEOs anode. In general, HEOs anode usually consists of electrochemically active components and inactive components. Such as Ti, Fe, Co, Ni, Mn, Cr, and Cu are usually regarded as the redox‐active elements for lithium storage, Zn can be alloyed with Li to form LiZn nanophase,^[^ ^79^ ^]^ providing additional lithium storage capacity, while Mg and Al are considered as the inactive components for preserving the small size of the active components, buffering volume change, and increasing the configuration entropy as well.^[^ ^76^ ^]^ Moreover, inactive components also can act as a barrier to hinder the agglomeration of the secondary particles and guarantee structural integrity.^[^ ^77^ ^]^

To compare different types of HEOs conveniently, **Table** **1** lists the microstructures, element composition, and electrochemical performance of the current HEO anode materials.

**Table 1:** A performance comparison of HEO anodes in LIBs.

HEO structure	Component	Initial capacity (0.1 A g^−1^)	Cycle retention^a)^	Rate capability	Synthesis	Reference
Spinel	(Mg_0.2_Ti_0.2_Zn_0.2_Cu_0.2_ Fe_0.2_)_3_O_4_	1261 mAh g^−1^	96.2% @ 800 cycles (2 A g^−1^)	272 mAh g^−1^ @ 2 A g^−1^	Solid‐state sintering	[57b]
Spinel	(Ni_0.2_Co_0.2_Mn_0.2_ Fe_0.2_ Ti_0.2_)_3_O_4_	900 mAh g^−1^	99.5% @ 100 cycles (0.1 A g^−1^)	343 mA h g^−1^ @ 2.5 A g^−1^	Solid‐state sintering	[80]
Spinel	(FeCoNiCrMn)_3_O_4_	1072 mAh g^−1^	90% @ 200 cycles (0.5 A g^−1^)	500 mA h g^−1^ @ 2 A g^−1^	Hydrothermal	[52]
Spinel	(Al_0.2_FeCoNiCrMn)_3_O_4_	1400 mAh g^−1^	40% @ 500 cycles (0.2 A g^−1^)	634 mA h g^−1^ @ 3 A g^−1^	Solid‐state sintering	[81]
Spinel	(FeCoNiCrMnZnLi)_3_O_4_	1049 mAh g^−1^	75% @ 100 cycles (0.1 A g^−1^)	173 mA h g^−1^ @ 2 A g^−1^	Solid‐state sintering	[82]
Spinel	(FeCoNiCrMn)_3_O_4_	967 mAh g^−1^	62% @ 1200 cycles (2 A g^−1^)	482 mA h g^−1^ @ 3 A g^−1^	Alloy oxidation	[83]
Spinel	(FeCuNiCrMn)_3_O_4_	720 mAh g^−1^	90% @ 150 cycles (0.5 A g^−1^)	340 mA h g^−1^ @ 2 A g^−1^	Hydrothermal	[84]
Spinel	(FeCoNiCrMn)_3_O_4_	750 mAh g^−1^	80% @ 5000 cycles (5 A g^−1^)	423 mA h g^−1^ @ 1 A g^−1^	Solution combustion	[74]
Spinel	(FeCuNiCrMn)_3_O_4_	900 mAh g^−1^	99% @ 250 cycles (0.5 A g^−1^)	451 mA h g^−1^ @ 2 A g^−1^	Hydrothermal	[85]
Spinel	(FeCoNiCrMn)_3_O_4_	1034 mAh g^−1^	67% @ 300 cycles (0.5 A g^−1^)	180 mA h g^−1^ @ 2 A g^−1^	Solid‐state sintering	[48a]
Spinel	(FeMgAlNiCr Mn)_3_O_4_	865 mAh g^−1^	99% @ 200 cycles (0.2 A g^−1^)	350 mA h g^−1^ @ 4 A g^−1^	Solution combustion	[76]
Spinel	(FeCoNiZnMnLi)_3_O_4_	1104 mAh g^−1^	84% @ 100 cycles (0.1 A g^−1^)	225 mA h g^−1^ @ 2 A g^−1^	Solid‐state sintering	[27]
Spinel	(FeCoNiCuZnMn)_3_O_4_	740 mAh g^−1^	86% @ 100 cycles (0.13 A g^−1^)	300 mA h g^−1^ @ 1.6 A g^−1^	Joule heating	[58]
Spinel	(FeZnNiCrMn)_3_O_4_	692 mAh g^−1^	70% @ 185 cycles (0.5 A g^−1^)	260 mA h g^−1^ @ 3 A g^−1^	Solid‐state sintering	[86]
Spinel	Li_1.8_(FeCoZnCrMn)_3_O*_x_*	400 mAh g^−1^	81% @ 300 cycles (0.5 A g^−1^)	146 mA h g^−1^ @ 1 A g^−1^	Sol‐gel	[67]
Spinel	(FeCoNiCrZn)_3_O_4_	1022 mAh g^−1^	99% @ 1000 cycles (1 A g^−1^)	220 mA h g^−1^ @ 30 A g^−1^	Sol‐gel	[87]
Rock salt	(MgCoNiCuZn)O	955 mAh g^−1^	97% @ 300 cycles (0.1 A g^−1^)	490 mA h g^−1^ @ 3 A g^−1^	Solid‐state sintering	[77b]
Rock salt	(Mg_0.8_CoNiCuZn)O	850 mAh g^−1^	53% @ 100 cycles (3 A g^−1^)	206 mA h g^−1^ @ 3 A g^−1^	Solid‐state sintering	[88]
Rock salt	(MgCoNiCuZn)O	480 mAh g^−1^	76% @ 300 cycles (0.2 A g^−1^)	100 mA h g^−1^ @ 3 A g^−1^	Electrospinning	[89]
Rock salt	(MgCoNiZn)_0.65_ Li_0.35_O	925 mAh g^−1^	85% @ 300 cycles (1 A g^−1^)	680 mA h g^−1^ @ 1 A g^−1^	Solid‐state sintering	[55]
Rock salt	(MgCoNiCuZn)O@Graphene	1225 mAh g^−1^	97% @ 1000 cycles (1 A g^−1^)	393 mA h g^−1^ @ 2 A g^−1^	Solid‐state sintering	[90]
Rock salt	(MgCoNiCuZn)O@PANI	823 mAh g^−1^	58% @ 3200 cycles (4 A g^−1^)	325 mA h g^−1^ @ 10 A g^−1^	Hydrothermal	[53]
Rock salt	(MgCoNiCuZn)O	500 mAh g^−1^	99% @ 300 cycles (0.2 A g^−1^)	180 mA h g^−1^ @ 3 A g^−1^	Nebulized spray pyrolysis	[91a]
Rock salt	(MgCoNiCuZn)O	450 mAh g^−1^	82% @ 600 cycles (0.2 A g^−1^)	280 mA h g^−1^ @ 0.5 A g^−1^	Nebulized spray pyrolysis	[92]
Perovskite	[(Bi,Na)_1/5_(La, Li)_1/5_(Ce, K)_1/5_Ca_1/5_Sr_1/5_]TiO_3_	130 mAh g^−1^	98% @ 300 cycles (1 A g^−1^)	59 mA h g^−1^ @ 3 A g^−1^	Solid‐state sintering	[26]
Perovskite	Li_0.1_(LiLaCaSrBa)Ti_0.9_Al_0.1_O_3_	92 mAh g^−1^	96% @ 100 cycles (0.1 A g^−1^)	37 mA h g^−1^ @ 1 A g^−1^	Solid‐state sintering	[91b]

^a)^ All the data are normalized according to the test data of the original literature.

### 4.2 Cathode

High entropy approach has become a prevalent strategy to design advanced cathode materials with higher energy density and superior cycle stability for the current LIBs and SIBs.^[^ ^18^, ^33^ ^]^ The advantages of HEO cathode materials are summarized as follows.

**Figure 5:**
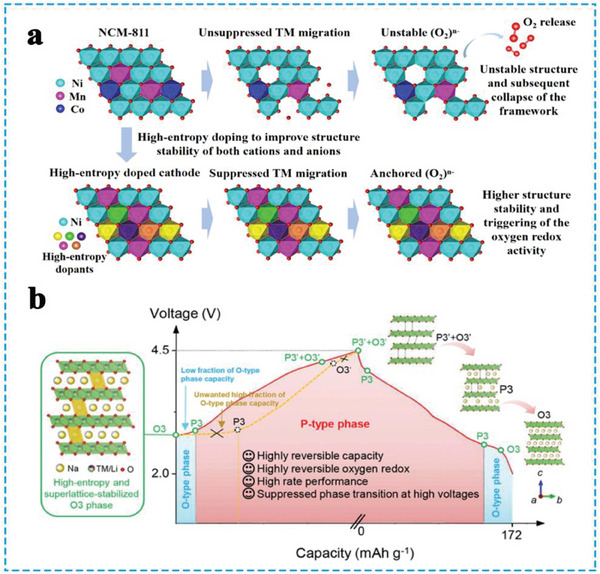
a) Schematic diagram of the cationic high‐entropy doping to stabilize the structure and achieve oxygen redox stability. Reproduced with permission.^[^ ^95^ ^]^ Copyright 2024, Wiley‐VCH. b) Schematic illustration of charge/discharge behaviors for high‐entropy and superlattice‐stabilized O3‐type layer oxide. Reproduced with permission.^[^ ^96^ ^]^ Copyright 2023, Wiley‐VCH.

1. Enhanced structural stability: In the realm of SIBs, extensive research has been conducted on layered oxides (Na*_x_* TMO_2_), which hold great promise as cathode materials. However, layered oxide cathodes inevitably face intricate phase transitions during the charge/discharge process, posing challenges in maintaining stability, especially in the state of sodium deficiency.^[^ ^93^ ^]^ High entropy layer oxide cathodes displayed superior cycling stability, because the lattice distortion effects break the Na^+^ ion/vacancy ordering, and the sluggish effects caused by various sizes of cations effectively restrain the phase transition behaviors during the charge/discharge process.^[^ ^94^ ^]^ High‐entropy approach has been implemented to address the stability issues faced by high‐Ni (NCM‐811) cathodes (**Figure** **5**a). High‐entropy cathode involves the random assignment of TM elements, and accommodates different local characteristics, effectively preventing TM ion migration, potential phase transitions, and anchoring (O2)^n−^ anions, which allows for the preservation of capacity contribution while resolving the dilemma of cationic structure stability, thereby ensuring long‐term cycling stability.^[^ ^34^ ^]^

**Figure 6:**
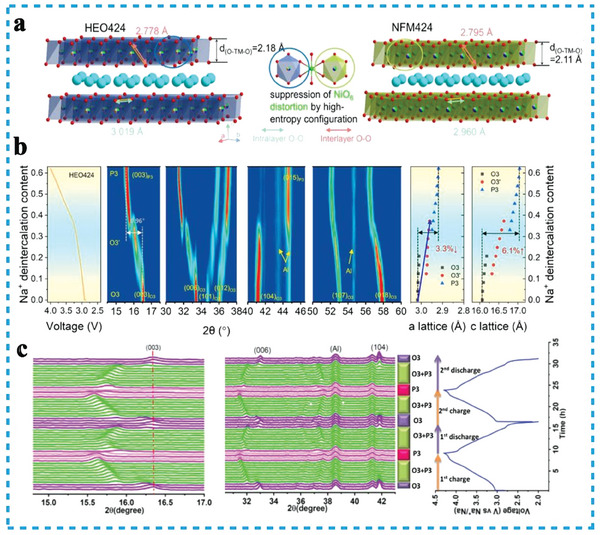
a) Schematic illustrations showing the TMO_2_ slabs in NFM424 and HEO 424 cathodes; b) structural evolution during the initial charge process of HEO424. Reproduced with permission.^[^ ^99^ ^]^ Copyright 2022, American Chemical Society. c) In situ XRD pattern of HE layer oxides and corresponding voltage profiles in the range of 2–4 V. Reproduced with permission.^[^ ^101^ ^]^ Copyright 2023, Wiley‐VCH.

In this context, high entropy TMO_2_ slabs play a crucial role in determining the crystal structure and properties. They not only act as redox centers for charge compensation but also serve as structural skeletons stabilizing the crystal framework. The dual functionality of high entropy TMO_2_ slabs enhances reliability and effectively addresses the aforementioned challenges. To further enhance reliability, especially under high charging voltages, great efforts have been made to tackle the undesired phase transitions and oxygen redox issues. For example, Yao et al. designed an HEO cathode Na_2/3_Li_1/6_Fe_1/6_Co_1/6_Ni_1/6_Mn_1/3_O_2_ for SIBs, wherein the TMO_2_ configuration entropy reaches 1.56 R and the doped lithium promotes the superlattice structure in the TMO_2_ slabs (Figure 5b). The high‐entropy O3‐type cathode material undergoes a rapid phase transition from O3 to P3 during the initial stage of the charging process, and presents a low fraction of O‐type phase capacity, which is advantageous for achieving high‐rate performance and long‐cycle stability.^[^ ^97^ ^]^ Moreover, to handle the deleterious phase transformations at deep‐desodiation state in O3‐type HEO cathode, Wang et al. elaborately designed Na‐deficient O3‐type Na_0.83_Li_0.1_Ni_0.25_Co_0.2_Mn_0.15_Ti_0.15_Sn_0.15_O_2−_ *_δ_* through manipulating the stoichiometric ratios of inactive cations (Li^+^, Ti^4+^) to optimize the TMO_2_ slabs configuration entropy from 1.26 R to 1.75 R, and achieve an impressive rate capability. Compared with Na_0.83_Li_0.1_Ni_0.25_Co_0.2_Sn_0.45_O_2−_ *_δ_*, the optimal HEO cathode displayed highly reversible redox reaction and O3‐P3‐O3 phase‐transition behavior, showed superior Na^+^ ion transport kinetics and reversible electrochemical reaction, achieved longer cycle life.^[^ ^98^ ^]^ Furthermore, Ding et al. constructed a high‐entropy configuration (1.83 R) in TMO_2_ slabs for NaNi_0.25_Mg_0.05_Cu_0.1_Fe_0.2_Mn_0.2_Ti_0.1_Sn_0.1_O_2_(HEO424) by substituting the NaNi_0.4_Fe_0.2_Mn_0.4_O_2_ (NFM424) cathode with divalent and tetravalent ions (**Figure** **6**a). The pure single‐phase structure was maintained throughout the initial charging and discharging process for the HEO424 cathode, implying successfully suppressed the hexagonal to monoclinic phase transition behavior, and formed a Na‐deficient O3’ phase (Figure 6b).^[^ ^99^ ^]^ In general, the transition from O3 to P3 phase accompanied by an O3’ phase (O3‐O3’‐P3), Joshi et al. presented a cobalt‐free high‐entropy cathode Na_0.9_Li_0.1_Ni_0.4_Fe_0.2_Mn_0.44_Ti_0.04_Mg_0.02_O_1.9_F_0.1,_ suppressed of the phase transition (O3‐O3’), maintained the structural integrity of the cathodes during sodiation/desodiation process (Figure 6c).^[^ ^100^ ^]^

**Figure 7:**
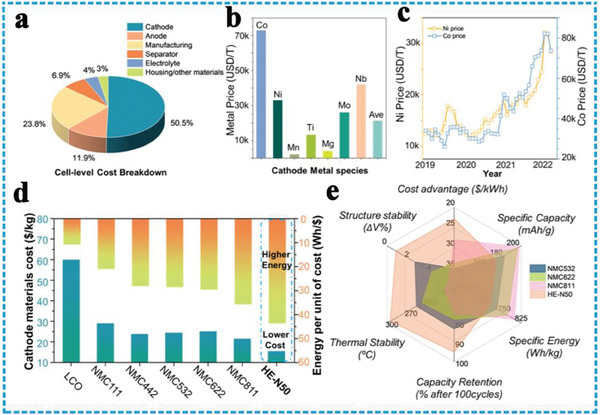
Battery cost comparison and multi‐dimensional evaluation of HEO cathode. a) Typical cell‐level cost breakdown of ternary NMC battery; b) price comparison of Ni, Co, Mn, and other alternative TM dopants; c) the price trend of Ni and Co since 2019; d) cathode materials cost and energy delivered per dollar of representative commercial NMC cathode; e) multi‐dimensional evaluation of HE‐N50, NMC‐532, NMC‐622 and NMC‐811. Reproduced with permission.^[^ ^104^ ^]^ Copyright 2023, Springer Nature.

**Figure 8:**
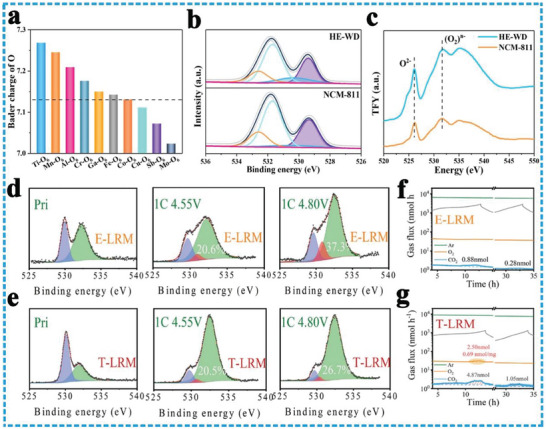
a) Bader charge of O in various TM‐O_6_ configurations; b) the O 2p XPS spectra and fitting results of HE‐WD and NCM‐811 in the charge state. c) O K‐edge XANES data collected from HE‐WD and NCM‐811 in the charged state. Reproduced with permission.^[^ ^95^ ^]^ Copyright 2024, Wiley‐VCH. d,e) O XPS evolution spectrum during the charging process of E‐LRM and T‐LRM; f,g) differential electrochemical mass spectrometry data of E‐LRM and T‐LRM. Reproduced with permission.^[^ ^107^ ^]^ Copyright 2022, Wiley‐VCH.

**Figure 9:**
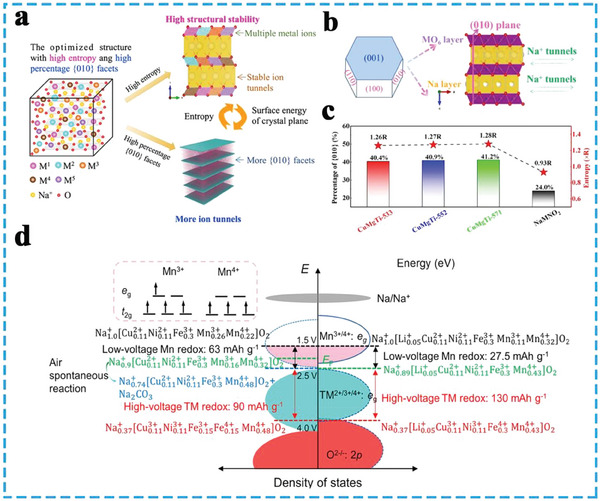
a) Design strategy of optimized P2‐type cathodes structure; b) schematic illustration of HEO microparticles with six {010} facets and two {001} facets; c) the percentage of {010} facets and configurational entropy of CuMgTi‐533, CuMgTi‐552, CuMgTi‐571, and NaMNO_2_ samples. Reproduced with permission.^[^ ^110^ ^]^ Copyright 2022, Springer Nature. d) Schematic illustration of the electronic structure adjustment of Na_0.9_Cu_0.11_Ni_0.11_Fe_0.3_Mn_0.48_O_2_ and Na_0.89_Li_0.05_Cu_0.11_Ni_0.11_Fe_0.3_Mn_0.43_O_2_. Reproduced with permission.^[^ ^94b^ ^]^ Copyright 2023, American Chemical Society.

1. Reduced reliance on rare elements: Cathode material plays a core role in the components for LIBs, which determines the comprehensive performance of the battery such as energy density, cycle life, and rate performance, and accounts for 50.5% of the total cell cost (**Figure** **7**a). The substitution of rare and expensive elements in cathode materials is a key concern due to the limited crustal reserves and geopolitical challenges associated with these elements.^[^ ^102^ ^]^ Aiming at solving the dilemmas of cobalt resource scarcity and the long‐standing safety and large volume change concerns (Figure 7b),^[^ ^103^ ^]^ HEOs offer the potential to reduce reliance on rare elements by employing a mixture of abundant transition metals, often avoiding or minimizing the need for rare and expensive elements. HEOs offer a platform for the development of durable and long‐lasting cathode materials. By leveraging the unique properties of HEOs, researchers can pursue the development of advanced cathode materials that offer sustainable alternatives to conventional rare elements‐based formulations. Xin et al. proposed the high entropy layered cathode LiNi_0.8_Mn_0.13_Ti_0.02_Mg_0.02_Nb_0.01_Mo_0.02_O_2_, exhibited an unprecedented zero volumetric change during Li^+^ ion de/intercalation, and realized zero strain and high capacity simultaneously.^[^ ^100^ ^]^ Afterward, Zhu et al. proposed a high entropy doping strategy by various low electronegativity cations dopant combinations replacing Co element and designed a high‐nickel Co‐free HEO cathode (LiNi_0.8_Mn_0.12_Al_0.02_Ti_0.02_Cr_0.02_Fe_0.02_O_2_), which has a higher capacity and cycle stability. This substitution can contribute to the development of more sustainable and cost‐effective energy storage technologies, particularly in the context of LIBs. With the rapid spread of rechargeable battery technology and the expansion of the commercial LIBs market, the price of Ni has risen rapidly as Co (Figure 7c). To achieve high specific energy and cycle stability, reduce the content of Ni, and eliminate the use of Co, Zhang et al. also successfully synthesized low‐Ni and Co‐free HEO cathode LiNi_0.5_Mn_0.43_Ti_0.02_Mg_0.02_Nb_0.01_Mo_0.02_O_2_ (HE‐N50), broke the frustrating trade‐off predicament between stability, cost and specific energy for the current commercial NMC (Ni, Mn, Co) cathodes (Figure 7d).^[^ ^104^ ^]^ Moreover, at the expense of negligible specific capacity, the HE‐N50 cathode demonstrates superior structural stability, better thermal stability, preferable capacity retention, and cost advantages than the commercial NMC532, NMC622, and NMC811 cathodes (Figure 7e).
2. Enhance the oxygen redox activity and improve lattice oxygen stability: It has been demonstrated that oxygen anions can participate in the charge compensation reaction in the Na^+^ ion (de)intercalation process and achieve extremely high energy density.^[^ ^105^ ^]^ Unfortunately, oxygen redox cathode materials often encounter irreversible oxygen loss and gas production side effects. The lattice oxygen in the layered oxide cathode usually undergoes the following two oxidation reactions: O^2−^ ⇋ (O2)^n−^ → O2. The overoxidation of oxygen anions in the second step induces the irreversible lattice oxygen loss.^[^ ^95^ ^]^ HEOs offer a unique opportunity for precise control over composition, allowing for the activation of oxygen redox activity and improvement in the stability of lattice oxygen.^[^ ^106^ ^]^ By introducing low‐electronegativity TM dopants, electron transfer in HEO materials can be induced, leading to an increase in the electron density of oxygen and thus enhancing redox activity. To further investigate this effect, Bader charge comparisons were performed using DFT calculations on various TMO_6_ configurations, and it was observed that TM dopants with relatively low electronegativity resulted in increased Bader charge (**Figure** **8**a). In addition, X‐ray photoelectron spectroscopy (XPS) and O K‐edge X‐ray absorption near‐edge structure (XANES) measurements confirmed that high‐entropy doped cathodes (HE‐WD) had a significantly higher content of (O2)^n−^ species compared to NCM‐811 cathodes, indicating enhanced reversible anion redox O2‐ ⇋ (O2)^n‐^ activity (Figure 8b,c). Xia's group also adopted a high entropy strategy to modify the typical Li‐rich cathode (Li_1.20_Mn_0.54_Ni_0.13_Co_0.13_O_2_, T‐LRM). To investigate the effect of high configuration entropy (*S*_conf_ = 1.51 R) on the interface and surface stability of HEO [Li_1.0_(Li_0.15_Mn_0.50_Ni_0.15_Co_0.1_Fe_0.025_Cu_0.025_Al_0.025_Mg_0.025_)O_2_, E‐LRM)], they used XPS to monitor the changes in the electronic structure of O anions in T‐LRM and E‐LRM samples at various charging stages (Figure 8d,e). The proportion of oxidized lattice oxygen in the E‐LRM reaches 37.3%, while only 26.7% for the T‐LRM at the charging state 4.8 V. It indicates that the oxygen loss from the anionic oxygen participation in charge compensation was effectively suppressed during the deep‐charged process. Furthermore, a comparison was made between T‐LRM and E‐LRM, regarding the gas production behaviors in the deeply delithiated‐state (Figure 8f,g). These findings confirmed that high entropy cathode materials exhibit a greater degree of local structural diversity, characterized by a variety of “Li‐O‐Li,” “Li‐O‐Mg,” and “Li‐O‐Al” combination configurations. This diversity plays a crucial role in regulating the charge compensation behavior of oxygen anions and effectively stabilizing the crystal structure.^[^ ^107^ ^]^
3. Expanded material design space: The presence of multiple components in HEOs expands the possibilities for designing cathode materials, allowing for the exploration of a wide range of chemical compositions and crystal structures. This expanded design space facilitates the discovery of new cathode materials with customized electrochemical properties, allowing for the optimization of parameters such as voltage, capacity, and rate capability. Rocksalt‐type HEO could be considered an unsealed platform to design cathode materials for lithium metal batteries. Facing abundant alternative TM candidates, designing HEOs cathode is a challenging endeavor due to the intricate interplay of each TM contribution to capacity and the need to prevent degradation during the cycling process. It is a compromise optimization strategy in the contradiction between capacity and cycle stability, by combining redox active elements and inactive elements. These important factors need to be considered when selecting elements, such as atomic size, chemical compatibility, electronegativity, and valence electron number.^[^ ^101^ ^]^ By adjusting the ratio of constituent metal cations, researchers can tailor the properties of HEO cathode materials to meet specific performance targets, such as high energy density, enhanced cycle life, and improved safety. Hu et al. first put forward high entropy layered O3‐type cathode NaNi_0.12_Cu_0.12_Mg_0.12_Fe_0.15_Co_0.15_Mn_0.1_Ti_0.1_Sn_0.1_Sb_0.04_O_2_ for SIBs, containing nine transition metal cations in the TMO_2_ slabs.^[^ ^34^ ^]^ Multi‐components are conducive to tolerating the change of local interactions during the Na^+^ (de)intercalation process. Afterward, Lun et al. designed high entropy rocksalt‐type cathode (Li_1.3_Mn^2+^_0.1_Co^2+^_0.1_Mn^3+^_0.1_Cr^3+^_0.1_Ti_0.1_Nb_0.2_O_1.7_F_0.3_) containing six TM species for LIBs, of which the compatibility of various TMs can be achieved by reducing short‐range ordered structure and rendering more random configurations, and simultaneously optimizing capacity, rate performance and energy density.^[^ ^108^ ^]^
4. Regulating crystal plane and electronic configuration: Manganese‐rich layered oxide (Na*_x_* MnO_2_) is particularly advantageous for SIBs due to its low cost, abundant availability, and high theoretical specific capacity. However, Na*_x_* MnO_2_ cathodes often experience significant volume variation during cycling, leading to rapid capacity decay. This volume variation also causes detrimental cationic migration and distortion of the oxygen network, resulting in voltage decay and poor kinetics.^[^ ^109^ ^]^ To circumvent these issues, Sun et al. utilized inert cations (Ti^4+^, Mg^2+^) to control the crystal facets of the cathode materials, resulting in a P2‐type Na_0.62_Mn_0.67_Ni_0.23_Cu_0.05_Mg_0.07_Ti_0.01_O_2_ cathode with increased exposure of active facets. This modification creates a stable host structure with more migration tunnels, enhancing the transport of Na^+^ ions (**Figure** **9**a). The arrangement of {010} planes in the modified cathode created an open structure that facilitated faster Na^+^ transport between the TMO_2_ layers (Figure 9b). The entropy‐tuned cathode exhibited a higher proportion of {010} facets (Figure 9c), providing more efficient pathways for Na^+^ transport and improved rate performance. Compared to the NaMNO_2_ cathode, the modified cathode demonstrated excellent long‐term cycling performance, showing minimal voltage fading and maintaining a robust structure for over 500 cycles at 1 C (120 mA g^−1^).^[^ ^110^ ^]^ Hu's group employed a high entropy strategy to modify the electronic configuration of Na*_x_* Cu_0.11_Ni_0.11_Fe_0.3_Mn_0.48_O_2_ cathode (Figure 9d). It was achieved by substituting low‐valence cations (Li^+^) to optimize the localized electronic structure and align the Fermi level appropriately. This substitution strategy facilitated the redox reactions of high‐voltage transition metals (TMs), maximizing their capacity contribution. As a result, the high‐voltage TMs (Cu, Ni, and Fe) contributed to 29% more capacity compared to the original cathode. This provides a paradigm for the realization of a high entropy strategy to design high‐energy‐density SIBs through synchronous regulation of electronic configuration and crystal plane.^[^ ^94b^ ^]^

According to the above discussion, the functions of common transition elements in high‐entropy layered oxides for SIBs will be summarized as follows. 1) Redox‐active metal species, Fe^3+^, Mn^2+^, Ni^2+^, Co^2+^, and Cu^2+^ as the main components providing the charge compensation; 2) Inactive cations, such as Mg^2+^, Zn^2+^, Al^3+^, and Ti^4+^stabilize the structural integrity, and suppress the phase transition; 3) Sn^4+^, Sb^5+^, Cu^2+^ and Fe^3+^ can increase the average discharge voltage; 4) Li^+^, Mg^2+^, and Zn^2+^ can trigger the anion redox of oxygen, and improve the capacity of cathode materials. The combination of these different kinds of transition metal elements enhances the diversity of layered oxide cathodes and creates more opportunities for developing preeminent performance SIBs. In addition, to further clarify the relationship between the composition, microstructure, and electrochemical performances, **Table** **2** lists the discharge performances of the high entropy layered cathode materials in SIBs recently reported.

**Table 2:** A comparison of high entropy layered cathodes in SIBs.

Structure	Cathode component	Voltage range [V]	Initial capacity [mAh g^−1^]	Cycle retention^a)^	Rate performance	Synthesis	Reference
O3	NaNi_0.2_Fe_0.2_Co_0.2_Mn_0.2_Ti_0.2_ O_2_	1.5–4.2	180	97% @ 100 cycles (0.1 C)	173 mAh g^−1^ (5 C)	Solid‐state sintering	[94a]
O3	Na_0.83_Li_0.1_Ni_0.25_Co_0.2_Mn_0.15_ Ti_0.15_Sn_0.15_O_2−_ *_δ_*	2.0–4.2	123	90.7% @ 100 cycles (0.24 A g^−1^)	83.3 mAh g^−1^ (1.2 A g^−1^)	Solid‐state sintering	[98]
O3	Na_0.8_Ni_0.2_Fe_0.2_Co_0.2_Mn_0.2_ Ti_0.2_O_2_	2.0–4.0	107	90% @ 100 cycles (0.05 C)	94 mAh g^−1^(5 C)	Solid‐state sintering	[111]
O3	NaNi_0.12_Cu_0.12_Mg_0.12_Fe_0.15_ Co_0.15_Mn_0.1_Ti_0.1_Sn_0.1_Sb_0.04_ O_2_	2.0–3.9	110	90% @ 200 cycles (0.5 C)	87.1 mAh g^−1^(5 C)	Solid‐state sintering	[34]
O3	NaCu_0.1_Ni_0.3_Fe_0.2_Mn_0.2_Ti_0.2_ O_2_	2.0–3.9	130	87% @ 100 cycles (0.1 C)	85 mAh g^−1^(5 C)	Sol‐gel	[112]
O3	NaNi_1/4_Co_1/4_Fe_1/4_Mn_1/8_Ti_1/8_ O_2_	2.0–4.0	128	97% @ 100 cycles (2 C)	38.6 mAh g^−1^(60 C)	Solid‐state sintering	[113]
O3	Na_2/3_Li_1/6_Fe_1/6_Co_1/6_Ni_1/6_ Mn_1/3_O_2_	2.0–4.5	171.2	64% @ 300 cycles (5 C)	78.2 mAh g^−1^(10 C)	Solid‐state sintering	[96]
O3	NaFe_0.2_Co_0.2_Ni_0.2_Ti_0.2_Sn_0.1_ Li_0.1_O_2_	2.0–4.2	130.6	72% @ 100 cycles (0.1 C)	80.8 mAh g^−1^(2 C)	Solid‐state sintering	[114]
O3	Na_0.94_Ni_0.29_Cu_0.1_Fe_0.16_Mn_0.3_Ti_0.15_O_2_	2.0–4.0	122	79% @ 300 cycles (0.5 C)	68 mAh g^−1^(4 C)	Solid‐state sintering	[115]
O3	NaMn_0.2_Fe_0.2_Co_0.2_Ni_0.2_Sn_0.1_Al_0.05_Mg_0.05_O_2_	1.5–4.2	152	60% @ 300 cycles (0.5 C)	69 mAh g^−1^(5 C)	Solid‐state sintering	[116]
O3	Na*_x_* Ti_1/6_Mn_1/6_Fe_1/6_Co_1/6_Ni_1/6_Cu_1/6_O_2_	1.5–3.9	141	77% @ 100 cycles (1 C)	39 mAh g^−1^(5 C)	Solid‐state sintering	[117]
P2	Na_0.667_Mn_0.667_Ni_0.167_ Co_0.117_Ti_0.01_Mg_0.01_Cu_0.01_ Mo_0.01_Nb_0.01_O_2_	1.5–4.5	197	76% @ 100 cycles (1 C)	111 mAh g^−1^(5 C)	Coprecipitation	[118]
P2	Na_0.6_Ti_0.2_Mn_0.2_Co_0.2_Ni_0.2_ Ru_0.2_O_2_	1.5–4.5	174	41% @ 400 cycles (0.02 A g^−1^)	39 mAh g^−1^(15 A g^−1^)	Solid‐state sintering	[29b]
P2	Na_0.75_Mn_0.55_Ni_0.25_Co_0.05_ Fe_0.10_Zr_0.05_O_2_	1.5–4.2	143	81% @ 100 cycles (0.1 C)	22 mAh g^−1^(10 C)	Sol‐gel	[119]
P2	Na_0.67_(Mn_0.45_Ni_0.18_Co_0.18_Ti_0.1_Mg_0.03_Al_0.04_Fe_0.02_)O_2_	1.5–4.6	102	86% @ 50 cycles (0.5 C)	/	Solid‐state sintering	[33]
P2	[Na_0.67_Zn_0.05_]Ni_0.22_Cu_0.06_ Mn_0.66_Ti_0.01_O_2_	2.0–4.2	123	92.7%@100 cycles (1 C)	91.5 mAh g^−1^(10 C)	Solid‐state sintering	[97]
P2	Na_0.62_Mn_0.67_Ni_0.23_Cu_0.05_ Mg_0.07_Ti_0.01_O_2_	2.0–4.3	148	75.4% @ 200 cycles (10 C)	82.6 mAh g^−1^(10 C)	Solid‐state sintering	[110]
O3/P2	Na_0.85_Li_0.05_Ni_0.25_Cu_0.025_Mg_0.025_Fe_0.05_Al_0.05_Mn_0.5_Ti_0.05_O_2_	2.0–4.2	122	90% @ 1000 cycles (10 C)	81.8 mAh g^−1^(10 C)	Solid‐state sintering	[120]

^a)^ All the data are normalized according to the test data of the original literature.

### 4.3 Electrolyte

**Figure 10:**
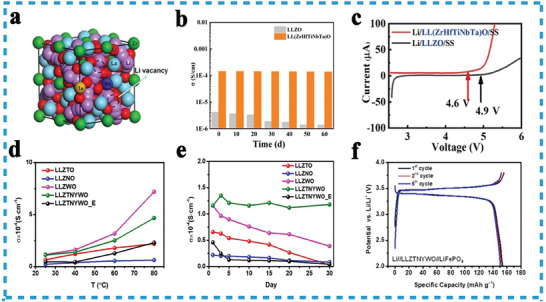
a) High‐entropy garnet structure; b) ionic conductivity as a function of exposure time observed in LL(ZrHfTiNbTa)O and LLZO pellets. c) LSV curves of Li/LL(ZrHfTiNbTa)O/stainless steel and Li/LLZO/stainless steel cells. Reproduced with permission.^[^ ^126^ ^]^ Copyright 2023, Springer. d) Comparison of all samples at different temperatures; e) comparison of all samples after exposure; f) galvanostatic charge/discharge curve of Li//LLZTNYWO//LiFePO_4_. Reproduced with permission.^[^ ^127^ ^]^ Copyright 2022, AIP.

With the congenital advantages of electronic conductivity and excellent mechanical properties, HEOs can be used as inorganic solid electrolytes or active fillers applied in the solid composite polymer electrolytes (SCE) in LIBs.^[^ ^121^ ^]^ Since Li or Na substituted HEOs stimulate the generation of a large number of oxygen vacancies according to the charge compensation mechanism, and exhibit superior ionic conductivity, providing the opportunity for all‐solid electrolytes development. Bérardan et al. first synthesized (Mg, Co, Ni, Cu, Zn)_1−_ *_x_* Li*_x_* O by substituting HEOs with monovalent Li^+^ cations, exhibited a high Li^+^ ionic conductivity that exceeds 10^−3^ S cm^−1^ at room temperature. This work hinted that ionic conductivity promotion could be achieved through the oxygen vacancies created by introducing monovalent cations in the compounds, which sheds light on the exploration of inorganic solid electrolytes with high ion conductivity.^[^ ^24^ ^]^ Currently, the most promising candidate for an ionically conductive solid electrolyte is Li_7_La_3_Zr_2_O_12_ (LLZO) with a garnet‐type crystal structure (**Figure** **10**a). To enhance its conductivity and structural stability, researchers have explored various strategies, such as substituting higher valence cations like Nb^5+^,^[^ ^122^ ^]^ Ta^5+^,^[^ ^123^ ^]^ Sb^5+^,^[^ ^124^ ^]^ and W^6+^,^[^ ^125^ ^]^ as well as lower‐valence cations like Y^3+^. These substitutions introduce more lithium vacancies and increase the concentration of Li^+^ ions, which effectively improve the conductivity and structural stability. Building upon these advantages, the garnet‐type HEOs can generate even more ionic vacancies and sufficient lithium‐ion concentration, offering the potential for composite solid‐state electrolytes (SSEs) with superior ionic conductivity. However, LLZO is susceptible to reactions with CO_2_ and H_2_O present in the ambient air, leading to a decay in its ionic conductivity upon exposure to air.^[^ ^28^ ^]^

To solve these issues, Kuo et al. introduced Ta, Nb, and W dopants at Zr sites, used the lower valence Y^3+^ to compensate for the decrease in Li^+^ concentration, synthesized the garnet‐type HEO, Li_6.4_La_3_Zr_0.4_Ta_0.4_Nb_0.4_Y_0.6_W_0.2_O_12_ (LLZTNYWO) by solid‐state reaction method. They used LLZTNYWO as the solid electrolyte in LIBs with a superior Li‐ion conductivity at 1.16 × 10^−4^ S cm^−1^, and remained constant for 60 days in the atmosphere without attenuation (Figure 10b), which revealed its excellent air stability.^[^ ^126^ ^]^ Han et al. also synthesized HEO Li_6.2_La_3_(Zr_0.2_Hf_0.2_Ti_0.2_Nb_0.2_Ta_0.2_)_2_O_12_ (LL(ZrHfTiNbTa)O) electrolyte by the solid‐state reaction method. The (LL(ZrHfTiNbTa)O) enabled electrochemically stable up to 4.6 V (Figure 10c) and suited for operating in high voltage solid LIBs.^[^ ^127^ ^]^ For the sake of air sensitivity and ion conduction decay of Li_7_La_3_Zr_2_O_12_, Kuo et al. proposed the garnet‐type HEOs, Li_6.4_La_3_Zr_0.4_Ta_0.4_Nb_0.4_Y_0.6_W_0.2_O_12_ (LLZTNYWO) by substituting Zr with Ta, Nb, Y, and W.^[^ ^126^ ^]^ It exhibits higher ionic conductivity compared to mono‐doped compounds at different temperatures (Figure 10d), maintains high ion conductivity for 30 days in the air atmosphere (Figure 10e), and displays desirable practicability in all‐solid‐state LIBs (Figure 10f).

**Figure 11:**
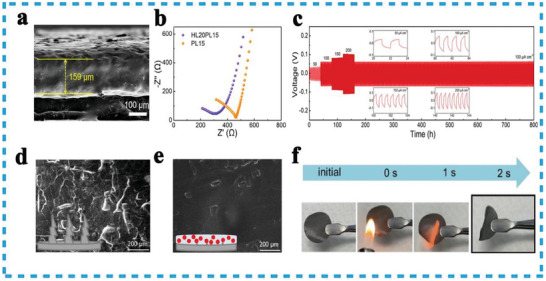
a) Side‐view SEM images of the HL20PL15 membrane; b) Nyquist plots of HL20PL15 and PL15 at 30 °C; c) cycling test with varying current densities. Reproduced with permission.^[^ ^121^ ^]^ Copyright 2022, American Chemical Society. d–f) The surface morphology of Li metal anode with various electrolytes of d) the liquid electrolytes; e) HEO/PEO SCE; f) fire‐resistance tests of HEO/PEO SCE. Reproduced with permission.^[^ ^129^ ^]^ Copyright 2023, Elsevier.

As good ionic conductors, HEOs are expected to be regarded as active fillers to improve the electrochemical properties of the polyethylene oxide (PEO) to form SCE.^[^ ^128^ ^]^ HEOs as the filler can preserve the electrolyte interface morphology, inhibit the growth of lithium dendrites, as well as, fire resistance. In their study, Chen et al. conducted a comparison of the surface morphologies of Li metal anodes using different electrolytes in the Cu/Li cell. They observed a significant formation of Li dendrites in the liquid electrolytes (**Figure** **11**d). However, when using HEO/PEO SCE, they found a relatively flat morphology (Figure 11e), reserving the structural integrity to prevent short circuits and enhance the safety of LIBs. To test the fire resistance, the researchers ignited the HEO/PEO SCE (Figure 11f) and observed high resistance to fire and signal suppression of inflammation, which was attributed to the addition of refractory HEO composites.^[^ ^129^ ^]^ Another study by Cai et al.^[^ ^121^ ^]^ involved the preparation of rock‐salt HEO with 20% Li ions, which was used as an active filler in a PEO‐based SCE. The HEO SCE was assembled into Li||Li symmetric batteries and cycled at 100 µA cm^−2^ for 800 h. The electrochemical impedance of the battery showed minimal variation, and the overpotential decrease remained at 65 mV during cycling at 100 µA cm^−2^. These results demonstrated the effectiveness of Li‐containing HEO as active fillers to improve the electrochemical performance of SCE, as well as their excellent compatibility with lithium‐metal anodes (**Table** **3**).

**Table 3:** The comparison of different HEO‐based electrolytes.

Types	Component	Structure	Ionic conductivity^a)^ [S cm^−1^]	Electrochemical window [V]	Reference
Inorganic solid electrolytes	Li_6.4_La_3_Zr_0.4_Ta_0.4_Nb_0.4_Y_0.6_W_0.2_O_12_	Garnet	1.16 × 10^−4^	2.5–6.0	[126]
	Li_6.925_La_2.95_Y_0.05_Zr_1.925_ Sb_0.075_O_12_	Garnet	3.2 × 10^−4^	2.5–6.0	[130]
	Li_7_La_3_Zr_0.5_Nb_0.5_Ta_0.5_Hf_0.5_ O_12_	Garnet	4.67 × 10^−4^	–	[28]
	(Ga_0.2_Li_5.75_La_2.5_Nd_0.5_Nb_0.65_Ce_0.1_Zr_1_Ti_0.25_O_12_)	Garnet	1 × 10^−4^	–	[131]
	Li_6.2_La_3_Zr_1.6_W_0.4_O_12_	Garnet	3.82 × 10^−5^	2.0–6.0	[126]
	Li_6.2_La_3_(Zr_0.2_Hf_0.2_Ti_0.2_Nb_0.2_Ta_0.2_)_2_O_12_	Garnet	1.42 × 10^−4^	2.0–4.6	[127]
Inorganic filler	Li_0.8_(CoCrFeMnNi)_2.2_O_4_	Spinel	1 × 10^−4^	2.0–4.9	[129]
	Li_0.2_Mg_0.15_Co_0.15_Ni_0.15_Cu_0.15_Zn_0.15_O_1‐_ *_x_*	Rock salt	3.44 × 10^−5^	2.0–4.5	[121]
	Li_0.25_Mg_0.15_Co_0.15_Ni_0.15_Cu_0.15_Zn_0.15_O_1‐_ *_x_*	Rock salt	8.9 × 10^−5^	2.8–4.85	[132]

^a)^ All the data on ionic conductivity at room temperature were collected.

### 4.4 Electrocatalysts for Li–S Batteries

The Li–S battery boasts a noteworthy theoretical energy density of 2600 Wh kg^−1^ and 2800 Wh L^−1^, positioning it as a promising contender for the next generation of high‐energy‐density battery systems.^[^ ^133^ ^]^ Extensive efforts have been dedicated to addressing challenges plaguing Li–S batteries, such as sluggish reaction kinetics and shuttle effects caused by confining sulfur within diverse host materials.^[^ ^134^ ^]^ These materials function to physically and chemically trap soluble lithium polysulfides (LiPSs) and expedite the electrochemical kinetics of sulfur redox.^[^ ^135^ ^]^ HEOs emerge as the promising sulfur‐host materials for Li–S batteries, offering distinctive advantages. First, the inherent thermodynamic stability and conductivity of HEOs significantly contribute to enhancing cycling stability and chemical stability within the working cell.^[^ ^136^ ^]^ Second, HEOs furnish an abundance of active sites with randomly occupied metal components, effectively anchoring lithium polysulfides (LiPSs) and facilitating the flow of electrons between cathode materials.^[^ ^137^ ^]^ Third, the synergistic effects of the multiple components present in HEOs enhance the absorptivity of Li_2_S_6_ and promote the electrochemical kinetics of the LiPSs conversion reaction.^[^ ^138^ ^]^

**Figure 12:**
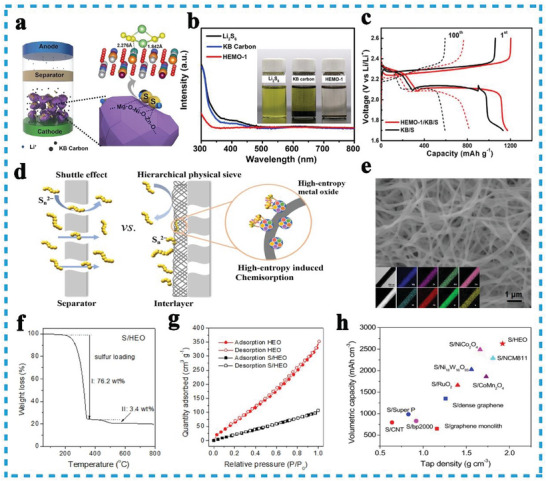
a) Schematic illustration of HEO as the chemical anchor of polysulfide in cathode for enhancing the performance of Li–S batteries. b) The absorption ability of Li_2_S_6_ solution comparison between (HEMO‐1) and KB carbon; c) discharge‐charge profiles of HEMO‐1 and KB at 0.1 C. Reproduced with permission.^[^ ^139^ ^]^ Copyright 2019, Elsevier. d) Schematic grapevine‐like HEO/CNFs interlayer in the Lithium‐sulfur battery. Reproduced with permission.^[^ ^140^ ^]^ Copyright 2022, Elsevier. e) HEO (Mg_0.2_Mn_0.2_Co_0.2_Ni_0.2_Zn_0.2_)Fe_2_O_4_ nanofibers SEM and elements mapping; f) TG curve; g) N_2_ adsorption/desorption isotherms curve; h) the volumetric capacity comparison between S/HEO and other sulfur/host composites. Reproduced with permission.^[^ ^73a^ ^]^ Copyright 2022, Wiley‐VCH.

As the pioneer practical case, Zheng et al. proposed HEO (Mg_0.2_Co_0.2_Ni_0.2_Cu_0.2_Zn_0.2_O, HEMO‐1) as the immobilizing mediator for LiPSs (**Figure** **12**a), effectively alleviated the shuttle effects, restricted the LIPSs and facilitated the redox reaction. By comparing the chemical adsorption of Li_2_S_6_ solution between HEMO‐1 and Ketjen Black (KB) carbon (Figure 12b), the absorption peak of Li_2_S_6_ in the visible light range disappeared after adding HEMO‐1, suggesting the strong adsorption of soluble LIPSs and stronger affinity. In addition, the discharge‐specific capacity and cycle retention have been effectively improved than KB carbon (Figure 12c).^[^ ^139^ ^]^

Considering the irregular bulk shape and exceptionally low surface area of HEO particles, the use of HEO nanofibers produced via electrospinning has emerged as a prominent solution for Li–S batteries due to their large specific surface area, high sulfur loading, high tap density, and abundant adsorption sites. The electrospinning method was employed to fabricate spinel HEO ((Mg_0.2_Mn_0.2_Co_0.2_Ni_0.2_Zn_0.2_)Fe_2_O_4_) nanofibers with a diameter range of 120–200 nm (Figure 12e). These nanofibers served as the electrocatalytic host, facilitating the conversion of soluble LiPS, and the resulting S/HEO electrode exhibited a substantial sulfur loading of 79.6 wt% and a large specific surface area of 154.7 m^2^ g^−1^(Figure 12f,g). These results demonstrated the robust entrapment and adsorption capabilities of HEO nanofibers, creating favorable conditions for the adsorption and catalysis of LiPS conversion.^[^ ^73a^ ^]^ Moreover, in comparison to porous carbon, graphene, and heavy oxides, HEO nanofibers hold significant potential for constructing high volumetric capacity sulfur cathodes (Figure 12h).

**Figure 13:**
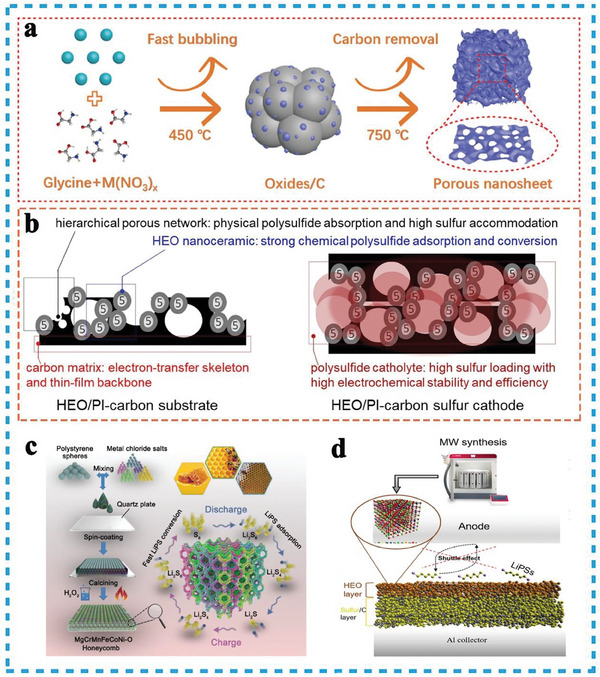
a) Schematic of the fabrication of mesoporous metal oxide nanosheets. Reproduced with permission.^[^ ^138^ ^]^ Copyright 2024, Wiley‐VCH. b) Schematic diagram of the HEO/PI‐carbon sulfur cathode. Reproduced with permission.^[^ ^136^ ^]^ Copyright 2023, Elsevier. c) Schematic of fabricating MgCrMnFeCoNi‐O honeycomb as a sulfur host. Reproduced with permission.^[^ ^141^ ^]^ Copyright 2023, RSC. d) Microwave‐assisted hydrothermal synthesized double‐coated cathode with HEOs for Li–S batteries. Reproduced with permission.^[^ ^142^ ^]^ Copyright 2022, Springer.

Except for HEOs nanofibers, high entropy sulfur host materials with high specific surface area and porous structure also have been reported recently. For example, Wang et al. adopt a glycine‐assisted strategy to synthesize the high crystallinity and remarkable porosity HEO LaFe_0.4_Co_0.2_Ni_0.2_Cu_0.2_O_3_ nanosheets (**Figure** **13**a), which as the catalyst for Li–S cell with an ultralong cycling life over 1500 cycles.^[^ ^141^ ^]^ To address the challenges of low sulfur‐loading capability and high polysulfide‐diffusion instability, Chung et al. employed the phase‐inversion method to fabricate an HEO/PI carbon‐sulfur cathode with (CrMnFeNiMg)_3_O_4_ nanoceramics. This approach resulted in a remarkable achievement of high sulfur loading (6–10 mg cm^−2^) and a content of 50 wt% (Figure 13b).^[^ ^136^ ^]^ Inherited the extraordinary physiochemical properties of HEAs furtherly enhance the catalytic activity, Zhou et al. enhanced the catalytic activity by developing a porous honeycomb structure using HEO MgCrMnFeCoNi‐O nanoparticles as a 3D sulfur host for Li–S batteries (Figure 13c).^[^ ^142^ ^]^ Despite notable progress in improving the capacity and cycle life of Li–S cells through these strategies, the intricate synthesis procedures have posed challenges to their practical implementation for commercialization. Colombo et al. have developed a dual‐layer cathode by sandwiching the sulfur/carbon active material between the aluminum current collector and HEO layer with microwave‐assisted hydrothermal synthetic methods. The implementation drastically reduced the processing time and the consumption of energy to obtain the intermediate powders (Figure 13d).^[^ ^143^ ^]^

Li–S batteries are still at an immature stage. Enhancing the performance of Li–S batteries demands innovative initiatives, necessitating advancements not only in the design of active materials but also in the comprehensive engineering of the entire cell composition.^[^ ^144^ ^]^ The enhanced catalytic activities of HEO materials stem from synergistic effects associated with the unique homogeneous solid solution structure, rather than a simple sum of components or a phase‐separated heterogeneous structure. The multi‐elemental synergy in HEO nanoparticles provides a variety of adsorption sites that are well suited to multi‐step tandem reactions requiring multifunctional catalysts.^[^ ^145^ ^]^ The diverse combination of metal cations in HEOs can create complex electronic structures with variable oxidation states and electronic configurations. Moreover, the presence of multiple metal cations in HEOs can lead to the formation of active sites with different chemical environments, which can enhance the Li_2_S_6_ adsorption and promote the conversion process.^[^ ^146^ ^]^ For comparison, we list these representative HEO electrocatalysts for Li–S batteries as shown in **Table** **4**.

**Table 4:** The performance comparison of sulfur cathodes with various HEOs electrocatalysts for Li–S batteries.

Cathode	Component	Structure	Sulfur loading [mg cm^−2^]	Reversible discharge capacity [mAh g^−1^]	Cycle retention^a)^	Reference
S@HEO/KB	(MgCoNiCuZn)O	Rock salt	1.2	664	54% @ 600 cycles (0.5 C)	[139]
S@HEO/CNT	(Mg_0.2_Mn_0.2_Co_0.2_ Ni_0.2_Zn_0.2_)Fe_2_O_4_	Spinel	2.8	879.6	63.5% @ 500 cycles (1 C)	[73a]
S@HEO/KB	(CrMnFeNiMg)_3_O_4_	Spinel	2.0–3.0	552	64% @ 300 cycles (0.1 C)	[136]
S@HEO/CNFs/KB	(Cu_0.7_Fe_0.6_Mn_0.4_Ni_0.6_Sn_0.5_)O_4_	–	3.4	907	48% @ 400 cycles (1 C)	[140]
S@HEO/CNT	La_0.8_Sr_0.2_(Cr_0.2_Mn_0.2_ Fe_0.2_Co_0.2_Ni_0.2_)O_3_	Perovskite	8.4	1038.6	68.8% @ 500 cycles (1 C)	[146]
S@HEO/CNT	(Ni_0.2_Co_0.2_Mn_0.2_Cu_0.2_Zn_0.2_)WO_4_	Orthorhombic	6.6	980	60% @ 500 cycles (1 C)	[73b]
S@HEO/KB	Co_0.08_Mn_0.08_Ni_0.08_Fe_1.96_ Mg_0.08_Nd_0.01_Gd_0.01_ Sm_0.01_Pr_0.01_O_4_	Spinel	1.4–2.0	698	76% @ 300 cycles (0.5 C)	[147]
S@HEO/KB	(Ni_0.2_Co_0.2_Cu_0.2_Mg_0.2_Zn_0.2_)O	Rock salt	4.4	1244	65.6% @ 800 cycles (0.5 C)	[148]
S@HEO nanosheets	LaFe_0.4_Co_0.2_Ni_0.2_Cu_0.2_O_3_	Cubic	1.5	1199.8	81% @ 150 cycles (0.2 C)	[141]
S@HEO/C layer	(Co_0.2_Cu_0.2_Mg_0.2_Ni_0.2_Zn_0.2_)O	Rock salt	1.0	1173	45% @ 500 cycles (0.2 C)	[143]

^a)^ All the data are normalized according to the test data of the original literature.

## 5 Outlook of Theoretical Calculation and Characterization

### 5.1 DFT Calculation

DFT has been widely applied in the field of HEOs to gain insights into their structural features, electronic conductivities, band gap, metallic‐oxygen binding energy, and thermodynamic properties. These calculations have provided insights into the phase stability, and transition behaviors under different conditions. It shows the following merits: 1) Calculating the total energy of different HEO crystal structures, we can identify the most stable configurations of HEOs and understand the factors that drive their stability; 2) Identifying the HEO electronic properties, such as band structures, density of states, and electronic transport properties, can provide valuable insights into the HEO electron conductivity, band gaps, and electronic behavior; 3) Providing a description of the ionic transport mechanisms, estimating the ionic diffusion energy barriers, and revealing the corresponding pathway dimensionality; 4) Investigating the thermodynamic properties, such as phase diagrams and phase stability. By calculating the formation energy and Gibbs free energy, researchers can determine the HEO stability of different phases and predict phase transitions under different temperatures and pressures.

**Figure 14:**
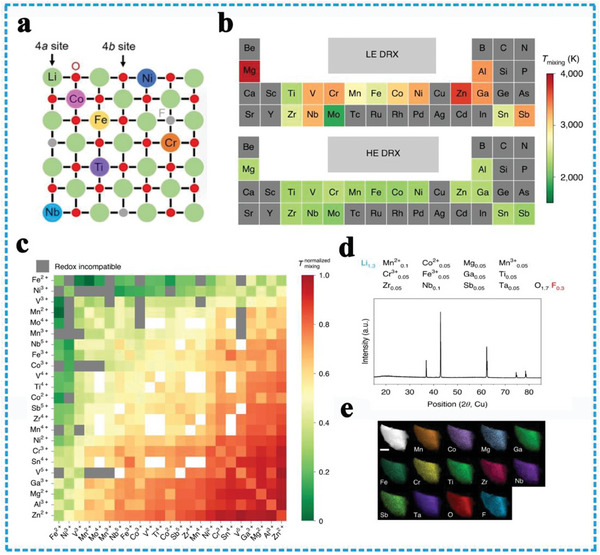
a) Schematic diagram of high entropy disordered rocksalt cathode structure. Reproduced with permission.^[^ ^149^ ^]^ Copyright 2023, Wiley‐VCH. b) Periodic table‐type heat map of mixing temperatures of different elements in low‐entropy and high‐entropy cation‐disordered rock salts; c) normalized mixing temperature of different TM species in HEO compounds; d) the XRD pattern of HEO containing 12 TM species; e) the STEM/EDS mapping. Reproduced with permission.^[^ ^108^ ^]^ Copyright 2021, Springer Nature.

The cation‐disordered rock‐salt oxide is a promising structural platform for designing desired high entropy electrode materials for LIBs, as it allows for accommodating a variety of heterovalent metal cations and flexible chemical components (**Figure** **14**a). In a study by Ceder et al., the mixing temperature of potential cation‐disordered rock‐salt compounds was estimated using Gibbs free energy calculations. It was found that low‐entropy compounds require higher mixing temperatures to achieve a random state compared to high‐entropy compounds (Figure 14b). To explore the possibilities of high‐entropy dual‐redox compounds, First‐principles DFT calculations were used to assess the coexistence of various oxides with different valence states (Figure 14c). The mixing temperature serves as an indicator of synthetic accessibility, with lower values indicating easier synthesis due to reduced thermal energy requirements. Herein, the compatibility of different transition metal pairs was represented on a scale from 0 (high compatibility) to 1 (incompatibility). By following the compatibility guidelines, an HEO compound consisting of 12 transition metal species was successfully synthesized in a single phase, with each element evenly distributed at similar concentrations. (Figure 14d,e).^[^ ^108^ ^]^

### 5.2 Machine Learning

**Figure 15:**
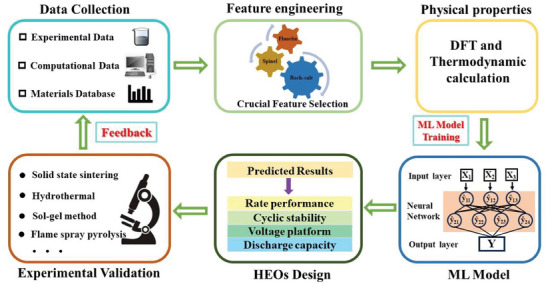
Schematic diagram of ML promoting the design of HEO materials. Reproduced with permission.^[^ ^150b^ ^]^ Copyright 2023, RSC.

Machine learning (ML) algorithms have the potential to greatly enhance the understanding and optimization electrochemical performance of HEO materials for rechargeable battery applications. ML process for HEOs mainly includes data collection, feature engineering, model training and selection, model deployment, experimental verification, and data set supplement (**Figure** **15**).^[^ ^150a^ ^]^ Besides, the DFT calculation data also can be regarded as the input source, and skip the feature engineering step. The representative ML algorithms including support vector machines, random forests, and neural networks, fulfill analyzation massive data and extract complex multi‐component relationships.^[^ ^151^ ^]^ These algorithms can be applied to predict HEO properties like phase stability, oxygen vacancy content, ionic conductivity, and chemical binding energy.^[^ ^152^ ^]^ These algorithms can be used to identify the above important features and parameters that influence their electrochemical performance, which is conducive to the design and synthesis of fresh HEO materials.

Additionally, ML also can be employed to predict and model the electrochemical performance of HEO materials under different conditions. Training the algorithm model on existing experimental data can be capable of simulating and predicting the performance under the same operating conditions. This can greatly accelerate the experimental exploration process, as it allows for rapid screening and optimization of different material compositions and structures.^[^ ^153^ ^]^ Furthermore, ML algorithms can assist in data‐driven decision‐making, analyze large datasets of experimental results and theoretical calculations, and predict the properties, helping researchers search for promising HEOs for specific applications. Considering crucial factors, such as performance, cost, and scalability. ML algorithms can aid in the selection of materials that are both technologically viable and economically feasible. Overall, the application of ML in HEO electrode materials holds tremendous potential for contributing to the discovery of new materials, optimization of the current HEO components, and accelerating the development of high‐performance energy storage devices.^[^ ^151a^ ^]^

The status of ML for HEOs can be described as promising but still in immature stages. It should be clarified that there are several challenges in using ML for HEOs. One major challenge is the lack of large and high‐quality datasets. HEOs are neonatal materials, and the experimental data for their properties is limited. This scarcity of data makes it challenging to train accurate ML models. Another challenge is the reliability, complexity, and high dimensionality of the current electrochemical data. These obstacles make it difficult to extract crucial features and patterns from the data, which affect the performance of ML models.

### 5.3 Characterization Techniques

There are many kinds of multi‐metal elements in the HEO materials, which play different roles in the process of charge and discharge process. Therefore, it is very necessary to develop advanced characterization techniques to clarify the roles of each metal in a redox reaction in the HEO materials. Several important characterization methods can be used to prove the HEOs, determine the valence state variations, coordinate environments, and monitor the electrochemical reaction process.

**Figure 16:**
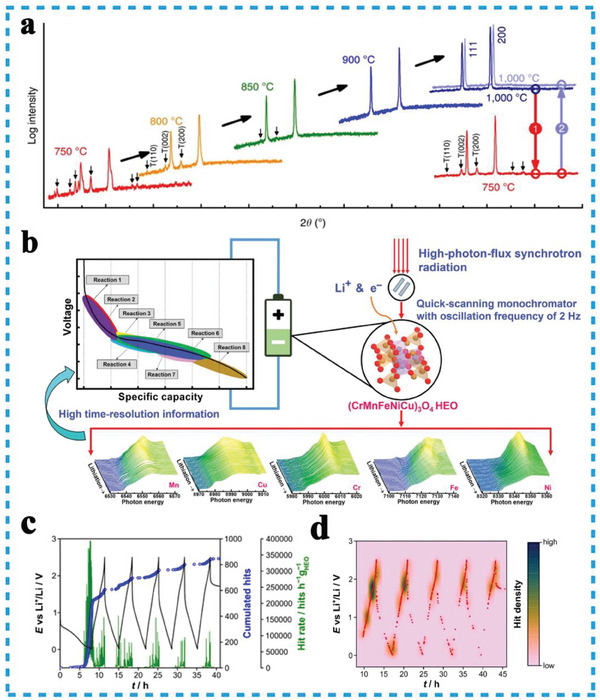
a) X‐ray diffraction patterns for HEO formulation. Reproduced with permission.^[^ ^7^ ^]^ Copyright 2015, Springer Nature. b) Using operando XAS to examine valence/coordination state variations, transition steps, redox sequence, reversibility, and redox overpotential in HEO electrode. Reproduced with permission.^[^ ^84^ ^]^ Copyright 2022, Wiley‐VCH. c) Voltage profile for the first five cycles (black) and corresponding cumulated hits (blue) and hit rate (green); d) contour plot of acoustic activity as a function of time and voltage. Reproduced with permission.^[^ ^154a^ ^]^ Copyright 2021, Springer.

1. X‐ray diffraction (XRD): XRD stands as an essential tool for confirming the single‐phase crystal structure of HEOs and is effective in tracking changes in the crystal structure at various temperatures. When combined with XRD Rietveld refinement, it allows for the determination of unit cell parameters for HEOs. It is noteworthy that a reversible phase transition with temperature has been demonstrated, showcasing the transformation from a single phase to a multiphase and back to a single phase (**Figure** **16**a). This serves as evidence for the distinctive characteristics of HEOs.^[^ ^7^ ^]^
2. X‐ray absorption spectroscopy (XAS): Employing bulk‐sensitive XAS provides insights into both electronic and structural properties at the bulk level. This technique serves as a direct means to ascertain the valence change and electronic structure of individual metal components within HEOs. It enables the determination of oxidation states of constituent elements and their interactions within the particle bulk.^[^ ^155^ ^]^ XANES spectra aid in comprehending the average oxidation state of transition metals by analyzing edge energy positions. XANES was performed to investigate the local coordination environment and the valance states of each transition metal cation in HEOs (Figure 16b). The Fourier transform of the Extended Edge X‐ray Absorption Fine Structure spectrum reveals periodicities or frequencies associated with specific electronic transitions and interactions occurring in HEOs.^[^ ^112^ ^]^
3. Acoustic emission (AE) technology: AE technology serves as a non‐destructive method applicable in real‐time, providing valuable insights into the behaviors of the HEO anode during electrochemical cycling. This technology enables the classification of acoustic signals, allowing for more accurate in situ experimental information. Monitoring the SEI formation and the mechanical degradation process becomes possible through this approach. For instance, Schweidler et al. applied AE technology to observe the conversion‐type HEO anode materials in LIBs. Their findings highlighted that the correlations between various electrochemical processes and acoustic events, particularly solid‐electrolyte interphase formation and chemo‐mechanical degradation, were most pronounced during the initial cycle (Figure 16c,d).^[^ ^154a^ ^]^

## 6 Challenges and Perspectives

**Figure 17:**
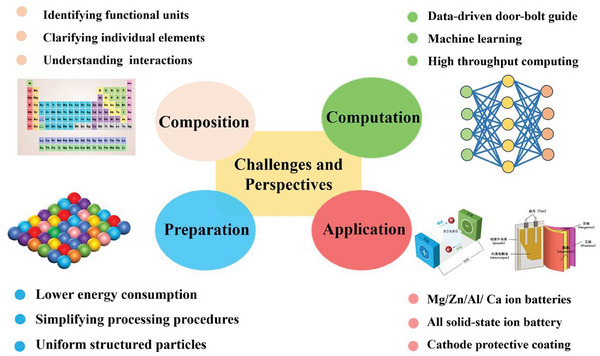
The challenges and perspectives for HEOs. Reproduced with permission.^[^ ^154b^ ^]^ Copyright 2024, Elsevier.

Although HEOs show great potential as the next generation of battery materials, they are still in the initial stage. The internal interaction mechanism, Artificial Intelligence approach, feasible environment‐friendly synthesis methods, and more dimensional application scenarios deserve further exploration. According to the current research situation, we give several views on the further development of HEMs in the field of rechargeable batteries. The main challenges and prospects associated with HEOs are outlined as follows (**Figure** **17**).

1. The advent of HEOs has ushered in fresh opportunities for customizing and improving material properties, offering a means to overcome the constraints of conventional energy materials. HEOs exhibit long‐range structural order within a periodic lattice framework, but their compositions are characterized by random disorder. While the extensive findings suggest that the attributes of HEOs can be tailored through composition regulation, the primary challenge at present lies in identifying functional units and comprehending the influence of individual elements in particular applications, as well as their interactions in composite effects. Future viable endeavors should tackle the intricacies of physics and chemistry in the quest for material correlations, understanding the effects of individual elements, and targeting specific applications.
2. It is imperative to establish a data‐driven door‐bolt guide for highly efficient and scientifically grounded strategies to replace the traditional trial‐and‐error approach in the laborious and time‐consuming process of deriving HEOs. Computational data‐driven material discovery, including ML and High throughput computing, is poised to revolutionize materials design and the discovery of HEOs. ML algorithm needs to be adjusted to be adaptive and optimize parameters in the manufacturing process, encompassing composition design, material preparation technology, and mechanistic research. Accelerating the development of ML models with extensive databases can enable the screening of vast combinations of structures and compositions, facilitating rapid element screening and the evaluation of constituent elements and their proportions. Consequently, in turn to aid in predicting properties and guiding future experiments to discover next‐generation HEO materials. To identify the most promising energy storage materials, several critical factors must be considered, including electrochemical stability, specific capacity, rate capability, cycling stability, and cost‐effectiveness.
3. The development of advanced, straightforward, and low‐energy techniques is of great importance for harnessing the potential of HEOs, particularly in achieving liquid‐phase synthesis under moderate conditions with minimal energy consumption. While significant strides have been made by researchers in establishing methods for HEOs synthesis. The existing techniques, such as FSP, atomization spray pyrolysis, and solid‐phase sintering processes, pose challenges related to intricate preparation procedures, structural heterogeneity, prolonged processing durations, and substantial energy consumption. The scientific community is actively engaged in the pursuit of advanced technologies and facilities simplifying the production of HEOs while minimizing energy consumption. As such, realizing this objective remains a challenging task that requires further exploration.
4. Apart from their application in Li/Na‐ion batteries, HEOs can find broader utility in the functional components of multivalent metal‐ion batteries, which encompass magnesium, aluminum, calcium, and zinc‐ion batteries. Entropy‐driven structural stability and enhanced ion diffusion kinetics, open new insights for developing advanced layered cathode materials for other rechargeable ion batteries as well. Further research and development are required to optimize the performance of HEOs and address any challenges associated with their practical implementation.

Additionally, HEOs possess multiple metal active sites and electron transfer capabilities suitable for Li–S batteries. Investigating the catalytic mechanisms of individual metal active sites and the synergistic effects of HEOs on polysulfides warrants further exploration. Enhancements in conductivity and the immobilization of polysulfides are also in need of further improvement. Furthermore, innovative and diversified approaches are needed to extend the development of HEO materials, for instance, using HEOs as the protective coatings for cathodes when operating in corrosive electrolyte environments due to their robust structure and chemical stability.

## 7 Conclusion

This review systematically summarized the developments of various HEO materials, including their classification, synthesis methods, and multiple applications in the field of rechargeable batteries. HEOs remain a captivating area of exploration within the realm of current electrode materials. Researchers have directed their efforts toward the synthesis and characterization of various HEO compositions, aiming to optimize their structural, electrical, and electrochemical attributes. Overall, the study of HEOs for energy storage and conversion is a dynamic and rapidly evolving field. Continuous advancements in experimental techniques, theoretical methods, and the application of machine learning are poised to the identification of novel HEO compositions that demonstrate outstanding energy storage capabilities.

This review presents a comprehensive exploration of HEOs, delving into their diverse structural features, the stabilizing effects driven by entropy, their electrical properties, and ionic conductivity. It encompasses an in‐depth examination of various synthesis methods, including a comparative analysis of their respective advantages and disadvantages. Furthermore, the paper investigates the current applications of HEOs in the context of LIBs and SIBs, offering valuable insights for future research in this burgeoning field. The review underscores the potential for artificial intelligence guidance and efficient synthesis strategies, envisioning the broadening of the compositional range and their growing significance in electrochemical energy storage and conversion applications.

## Conflict of Interest

The authors declare no conflict of interest.
